# Controlling Electrode–Electrolyte Interactions to Enhance Capacitance

**DOI:** 10.1021/jacs.5c20988

**Published:** 2026-04-13

**Authors:** Jamie W. Gittins, Chloe J. Balhatchet, James Hill, Teedhat Trisukhon, Malina Seyffertitz, Seung-Jae Shin, Yashna Khakre, Kangkang Ge, Thomas Kress, Smaranda C. Marinescu, Aron Walsh, Oskar Paris, Ieuan D. Seymour, Alexander C. Forse

**Affiliations:** † Yusuf Hamied Department of Chemistry, 2152 University of Cambridge, Lensfield Road, Cambridge CB2 1EW, U.K.; ‡ Wolfson Catalysis Centre, Department of Chemistry, University of Oxford, South Parks Road, Oxford OX1 3QR, U.K.; § Chair of Physics, Department Physics, Mechanics and Electrical Engineering, 27268 Montanuniversität Leoben, Franz-Josef-Straße 18, Leoben 8700, Austria; ∥ School of Energy and Chemical Engineering, Ulsan National Institute of Science and Technology (UNIST), Ulsan 44919, Republic of Korea; ⊥ Department of Chemistry, 5116 University of Southern California, Los Angeles, California 90089, United States; # CIRIMAT, UMR CNRS 5085, 27090 Université de Toulouse, Toulouse 31062, France; ∇ Thomas Young Centre & Department of Materials, 4615 Imperial College London, London SW7 2AZ, U.K.; ○ Advanced Centre for Energy and Sustainability (ACES), Department of Chemistry, 1019 University of Aberdeen, Meston Walk, Aberdeen AB24 3UE, U.K.

## Abstract

Understanding how ions interact with electrode surfaces at the molecular level is essential for improving the performance of energy storage devices and electrocatalysts. However, progress has been limited by the structural disorder and poorly defined surface chemistries of conventional carbon-based electrodes. In this work, we use layered metal–organic frameworks (MOFs) as model systems to investigate how different functional groups influence electric double-layer capacitance. We find that electrodes with deprotonated M–O and M–S groups exhibit significantly enhanced capacities with alkali metal cations, most notably Li^+^, compared to tetraethylammonium (TEA^+^), while no enhancement is observed for MOFs with protonated M–NH groups. The largest capacity increase is seen for MOF electrodes with metal–hydroxy linkages paired with Li^+^ electrolytes, which we attribute to strong Li–O interactions and improved charge screening. This mechanism is supported by solid-state nuclear magnetic resonance spectroscopy experiments and molecular simulations, which reveal specific Li^+^ binding at oxygen-rich sites, while operando X-ray techniques rule out cation intercalation as a contributing factor. Overall, these results highlight a chemically tunable strategy for enhancing charge storage in porous electrodes and offer new insights into how surface functionality impacts electric double-layer behavior.

## Graphical abstract

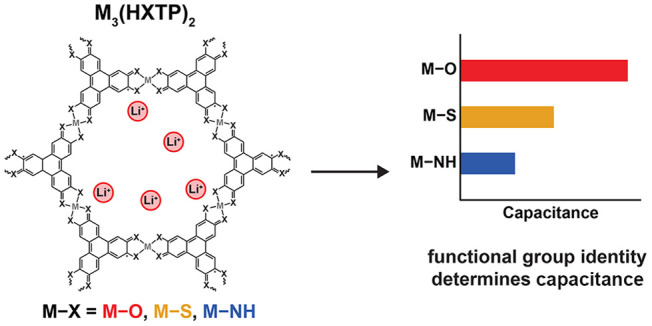

## Introduction

The structure of the electric double-layer, the interfacial region where electrolyte ions accumulate at a charged surface, is central to a wide range of electrochemical technologies, including energy storage, electrocatalysis, and sensing.^1–3^ A clear understanding of how electrode structure influences ion adsorption and charge separation is therefore essential for designing materials with improved performance. Functional groups on the electrode surface can modulate ion adsorption, charge transfer kinetics, and overall device performance.^4,5^ Porous carbons are the most widely used electrode materials in supercapacitors, but their disordered and amorphous nature make it difficult to attribute performance to specific surface chemistries (Figure 1a).^6–16^ To address this challenge, a number of studies have employed model carbon systems, including graphite, graphene-based materials, and chemically functionalized carbons, to probe how ion size, solvation, and surface chemistry influence electric double-layer structure and capacitance.^7,17,18^ These studies have begun to reveal how well-defined chemical functionalities affect ion adsorption and charge storage at carbon–electrolyte interfaces.

**Figure 1:**
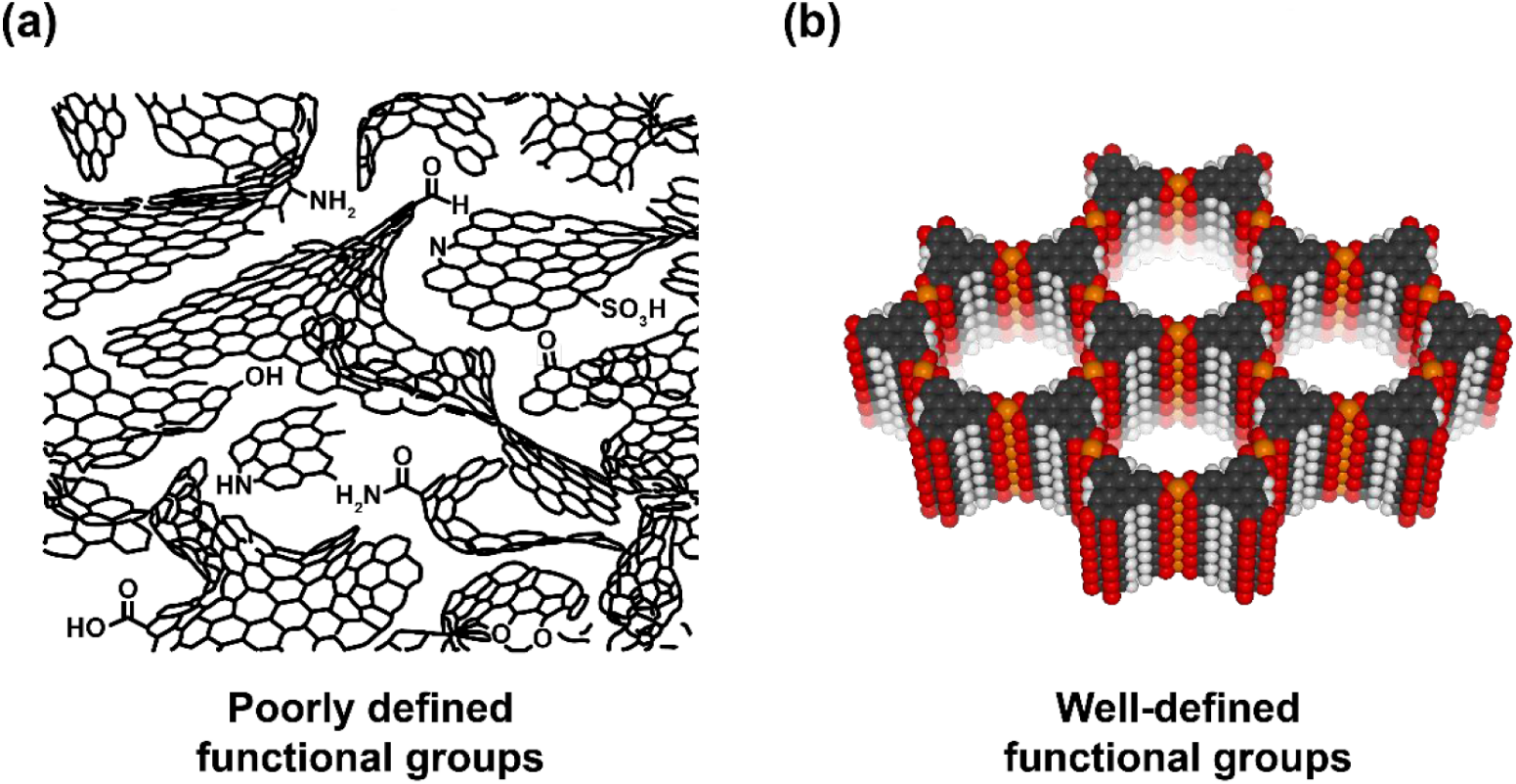
(a) Schematic representation of a porous carbon structure, consisting of disordered, curved graphene-like fragments, and slit-like pores. These materials contain a broad distribution of surface functional groups, making structure–performance studies challenging to perform and interpret. Adapted from Harris.^19^ (b) Generalized structure of a hexa-substituted triphenylene-based layered MOF, M_3_(HXTP)_2_, where M is the metal node and X is the coordinating heteroatom (e.g., O, S, or NH). In contrast to porous carbon materials, layered MOFs are crystalline and have uniformly distributed and well-defined pore functionalities, facilitating structure–performance studies.

Layered metal–organic frameworks (MOFs; Figure 1b) can be used to further address this challenge.^20^ These materials consist of stacked two-dimensional π-d conjugated layers, forming a honeycomb-like architecture that is porous, electronically conductive, and intrinsically ordered. Several layered MOFs have already been used as electrode materials in supercapacitors, with some exhibiting specific capacitances on par with or even exceeding those of porous carbon materials.^21–27^ Their structures are also tunable, and changing the metal node and ligating group allows the pore functionality to be systematically varied, facilitating structure–performance studies.

Previous studies have shown that modifying either the pore chemistry or electrolyte cation in layered MOFs can significantly enhance electrochemical performance, for example by introducing nitrogen into the backbone, altering the interlayer spacing, or switching the electrolyte cation from TEA^+^ to Li^+^.^28–31^ However, the mechanisms underlying these improvements remain poorly understood due to a lack of direct insights into ion-binding sites. Understanding these interactions is essential for the rational design of high-performance electrode materials. While MOFs are used primarily as model systems to understand electrode–electrolyte interactions, the insights gained from such a study may guide carbon electrode design, with similar ion–functional group interactions occurring at functionalized sites in amorphous carbon electrodes.

Here, we investigate how variations in metal–ligand bonding functionalities influence electric double-layer structure and charge storage in layered MOFs. Using a family of structurally related frameworks with different functional groups and a wide range of alkali metal and organic electrolytes, we probe how functional group identity and cation properties influence ion adsorption and charge storage. To understand the molecular origin of these effects, we combine electrochemical measurements with solid-state nuclear magnetic resonance (NMR) spectroscopy, computational results, and operando X-ray techniques. Our results reveal how local pore chemistry governs ion binding and double-layer structure in conductive MOFs, providing molecular-level insight into how electrode–electrolyte interactions can be tuned through pore functional group chemistry.

## Results and Discussion

### Impact of Cation Identity

Cu_3_(HHTP)_2_ (HHTP = 2,3,6,7,10,11-hexahydroxytriphenylene) was selected as a model electrode material due to its well-characterized electrochemical behavior, simple synthesis, and well-defined pore chemistry (Figure 2a; SI Figures S1–S3).^27,33–35^ To probe how electrolyte cations influence charge storage in Cu_3_(HHTP)_2_, symmetric two-electrode supercapacitor cells were assembled using 1 M bis­(trifluoromethanesulfonyl)­imide (TFSI^–^) in acetonitrile electrolytes with a range of cations: TEA^+^, Li^+^, Na^+^, and K^+^. All systems exhibited quasi-rectangular cyclic voltammograms (CVs) up to a cell voltage of 0.6 V, indicative of double-layer capacitive behavior (Figure 2b; SI Figures S4, S5; see SI Figure S5 for more details on the stable double-layer voltage window for each Cu_3_(HHTP)_2_–electrolyte system). All electrochemical measurements were performed on independently assembled cells prepared from separate MOF batches, with two or three independent cells measured for each MOF–electrolyte system depending on material availability. Excitingly, electrolytes containing alkali metal cations (Li^+^, Na^+^, and K^+^) exhibited significantly higher capacities than TEA^+^, as indicated by the larger CV areas, with Li^+^ showing the greatest enhancement (Figure 2b). Specific capacities measured at 0.05 A g^–1^ are summarized in Figure 2c (SI Figures S6, S7; SI Table S1). This behavior resembles that observed previously in other layered MOF systems.^30,31^ Three-electrode measurements with Li^+^ showed only minor differences in capacity upon positive and negative charging, confirming that the capacity enhancement is independent of electrode polarization (SI Figure S8). The open circuit potential was used as a reference potential for this measurement as the potential of zero charge (PZC) could not be identified for this MOF (SI Figure S9).

**Figure 2:**
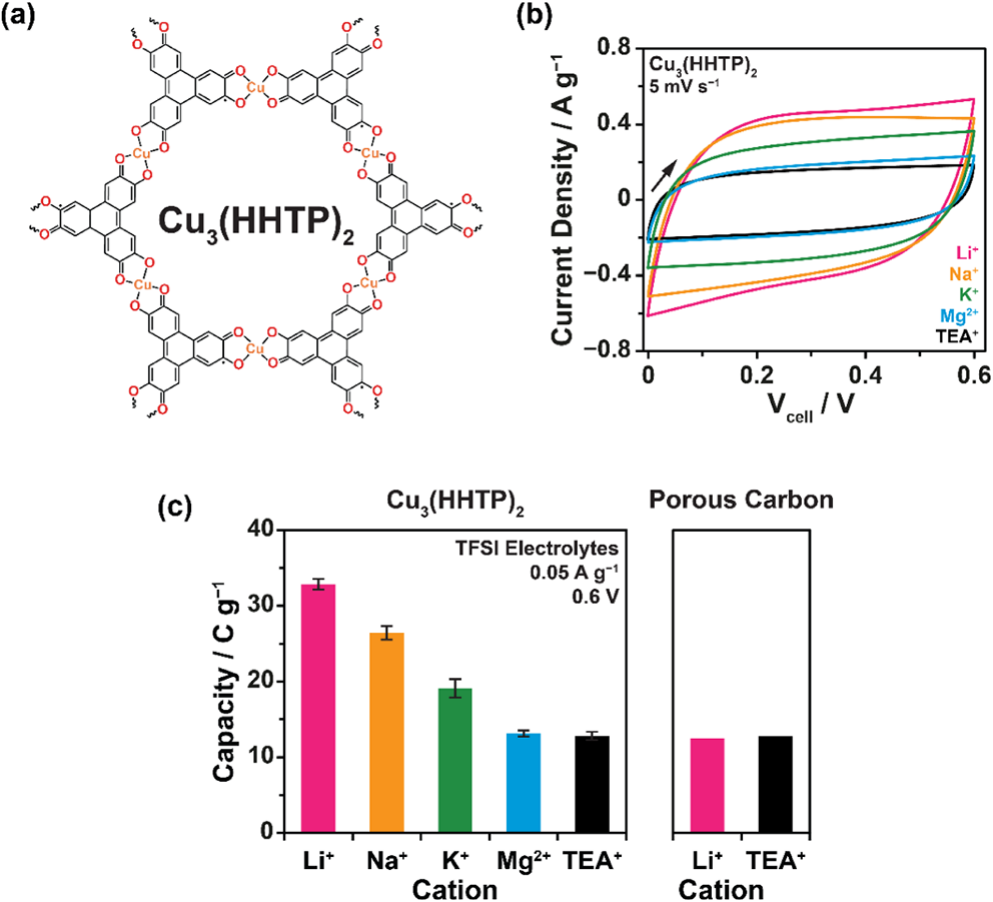
(a) Schematic showing the structure of the layered MOF Cu_3_(HHTP)_2_. (b) CVs obtained at a scan rate of 5 mV s^–1^ up to 0.6 V from two-electrode symmetric supercapacitors assembled with Cu_3_(HHTP)_2_ electrodes and 1 M solutions of LiTFSI, NaTFSI, KTFSI, TEATFSI and Mg­(TFSI)_2_ in acetonitrile electrolytes. (c) Specific capacity values for Cu_3_(HHTP)_2_ (left panel) and the porous carbon YP50F (right panel) with the same 1 M TFSI-based acetonitrile electrolytes, calculated from GCD experiments performed on symmetric two-electrode supercapacitor cells at a current density of 0.05 A g^–1^ when charging to a cell voltage of 0.6 V. Error bars for Cu_3_(HHTP)_2_ measurements represent measurements from at least two independent cells per system. Data for YP50F was obtained from one measurement for each electrolyte. Specific capacities are reported rather than capacitances, as recommended for systems with nonideal GCD behavior.^32^

For comparison, YP50F, a widely used commercial activated carbon, was tested under identical conditions to Cu_3_(HHTP)_2_. It was included as a representative porous carbon benchmark to enable qualitative comparison with a conventional activated carbon electrode material. YP50F was selected due to its widespread use in supercapacitor research and well-documented electrochemical performance.^36^ While the pore structure of YP50F differs from that of Cu_3_(HHTP)_2_, with a broader distribution of pore sizes and a higher overall surface area (SI Figure S10), it also crucially lacks the crystalline structure and well-defined coordination environments of layered MOFs. Instead, YP50F contains a broad and poorly defined distribution of surface functional groups (SI Figure S11). Interestingly, no capacity enhancement was observed in YP50F, with nearly identical capacities for both TEA^+^ and Li^+^ electrolytes (Figure 2c; SI Figure S12). This supports that the well-defined M–O functional groups of Cu_3_(HHTP)_2_ are critical for the observed capacity enhancements.

A clear trend was observed with Cu_3_(HHTP)_2_, with Li^+^ showing the greatest increase in capacity relative to TEA^+^ (2.5×), followed by Na^+^ (2.1×), and then K^+^ (1.5×) (Figure 2c). These differences likely arise from a balance between ion solvation in the bulk electrolyte, the energetic cost of partial desolvation upon approaching the electrode surface, and the strength of the resulting electrode–electrolyte interactions within the confined MOF pores. Partial desolvation of the alkali metal cations can occur in the MOF pores, allowing direct interaction with the oxygen-containing M–O functional groups of Cu_3_(HHTP)_2_. Li^+^ is expected to form particularly strong interactions with these functionalities due to its smaller ion size and stronger Lewis acidity. These interactions can reduce the effective double-layer thickness and enhance charge screening within the pores, enabling more efficient ion packing and increased capacity. The observed behavior is therefore most consistent with electric double-layer charging involving specific ion adsorption rather than a redox-like process.

This behavior differs from trends often reported for graphitic carbon electrodes, where ion adsorption is largely governed by electric double-layer formation and adsorption energetics depend strongly on ion desolvation and confinement effects.^37–39^ In such systems, larger and more weakly solvated cations can sometimes adsorb more favorably following partial desolvation at graphitic interfaces or within subnanometer pores. In contrast, the layered MOFs studied here contain chemically well-defined oxygen coordination environments that can stabilize specific cation–surface interactions. As a result, the balance between desolvation and adsorption is shifted toward smaller cations that interact more strongly with these functional groups. Cation-dependent behavior has also been reported for activated carbon electrodes with aqueous alkali metal electrolytes, although the direction of this effect is not universal. This variability suggests that the underlying mechanism in carbon materials remains unclear and may depend sensitively on pore structure, ion hydration, and electrolyte composition.^40–43^

To probe this interpretation further, additional measurements were performed with Mg­(TFSI)_2_. Interestingly, no improvement in the performance of Cu_3_(HHTP)_2_ was observed with Mg^2+^ compared to TEA^+^ (Figure 2b, 2c; SI Figure S13). This is consistent with the much stronger solvation of Mg^2+^ in acetonitrile, with previous work reporting a desolvation energy of approximately 491 kJ mol^–1^ for Mg^2+^ in acetonitrile, significantly higher than that of both Li^+^ (190 kJ mol^–1^) and Na^+^ (137 kJ mol^–1^).^44,45^ These findings support the previous hypothesis that partial desolvation is required for favorable electrostatic interactions between the cations and the hydroxy-functionalized pore surface. Therefore, despite its high ionic charge, the stronger solvation of Mg^2+^ likely hinders partial desolvation and prevents strong interactions with the M–O functional groups of Cu_3_(HHTP)_2_, resulting in no capacity enhancement. This suggests that other strongly solvated multivalent ions may be less able to form favorable interactions despite their higher formal ionic charges.

These results highlight the importance of considering the balance between ion solvation, the energetic cost of partial desolvation, and the strength of the resulting MOF–ion interactions when designing high-performance supercapacitor electrodes. While smaller cations such as Li^+^ can enhance performance in the present system, this behavior depends on their ability to partially desolvate and interact favorably with the oxygen-containing M–O functional groups in the MOF pores. More broadly, these results emphasize the central role of electrode–electrolyte interactions in determining electric double-layer structure and capacitance in nanoporous materials. However, although the capacity enhancement observed with Li^+^ electrolytes is substantial, the smaller electrochemical stability window of Cu_3_(HHTP)_2_ with these electrolytes limits the resulting device-level energy density, highlighting the importance of simultaneously optimizing both capacitance and voltage window in conductive MOF electrodes.

### Influence of Functional Groups

Having established that replacing TEA^+^ electrolytes with alkali metal electrolytes significantly enhances the electrochemical capacity of Cu_3_(HHTP)_2_, particularly with Li^+^, the effect of pore functionality on charge storage was investigated. Four additional structurally related frameworks were synthesized: (i) Zn_3_(HHTP)_2_, which retains the hydroxy (−O) ligating groups of Cu_3_(HHTP)_3_ with Zn metal nodes; (ii) Co_3_(THT)_2_ (THT = triphenylene-2,3,6,7,10,11-hexathiol), which has thiol (−S) ligating groups and Co metal nodes; (iii) Ni_3_(HITP)_2_ (HITP = 2,3,6,7,10,11-hexaiminotriphenylene), with imino (−NH) ligating groups and Ni metal nodes; and (iv) Cu_3_(HITP)_2_, which maintains the Cu metal node of Cu_3_(HHTP)_3_ but with imino (−NH) ligating groups (Figure 3a). These MOFs were selected as they have been successfully synthesized previously,^46–49^ and they enable a systematic investigation into how different pore functional groups influence performance.

**Figure 3:**
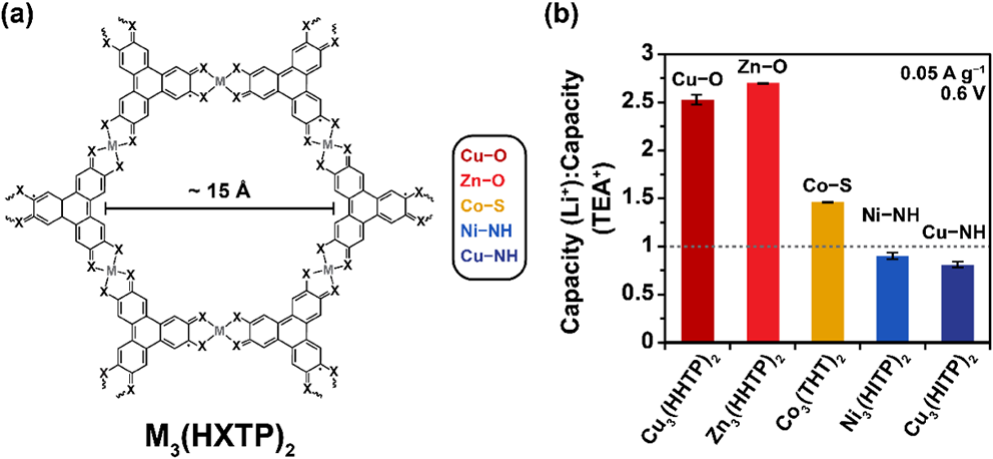
(a) Schematic representation of the structure of triphenylene-based layered MOFs, with the different combinations of metal node and ligating group used in this work summarized on the right. (b) Capacity ratios (capacity­(LiTFSI)/capacity (TEATFSI)) in 1 M acetonitrile electrolytes for a series of layered MOFs with different combinations of metal node and ligating group, calculated from GCD experiments on symmetric two-electrode supercapacitor cells at a current density of 0.05 A g^–1^ when charging to a fixed voltage of 0.6 V. Error bars represent measurements from at least two independent cells and MOF batches per system.

XRD analysis confirmed the successful synthesis of each layered MOF, with diffraction patterns aligning well with simulated data for an eclipsed AA-stacked framework (SI Figure S14). Interestingly, a shift in the (001) stacking peak to a higher value of 2θ was observed for HHTP-based MOFs compared to the other MOFs studied in this work, indicating a slightly smaller interlayer spacing (∼3.18 Å compared to ∼3.34 Å). In addition, minor differences in both peak intensity and peak broadness suggest variations in sample quality and crystallinity between the different layered MOFs. Porosity and surface area measurements via 77 K N_2_ isotherms further revealed significant differences in nitrogen-accessible surface areas and porosities across the MOFs (SI Figures S15, S16; SI Table S2), despite similar nominal pore sizes, likely due to variations in sample defects or incomplete sample activation.

Cyclic voltammetry confirmed stable capacitive behavior with predominantly double-layer charge storage for all systems up to 0.6 V (SI Figure S17; see SI Figures S18–S20 and SI Table S3 for a discussion of stable voltage ranges). However, differences in porosity and surface area between the different MOFs result in different accessible electrode surface areas available for double-layer formation, leading to significant variation in their absolute electrochemical capacities (SI Figure S21). To circumvent this, performance was evaluated for each MOF by comparing its capacity in TEA^+^ and Li^+^ electrolytes. This allowed the assessment of relative performance trends and helped to isolate the role of pore functionality in charge storage.

Excitingly, clear variations in capacity enhancement with Li^+^ were observed when the pore chemistry of the layered MOF was varied (Figure 3b; SI Figure S22 and SI Table S4). For layered MOFs with hydroxy ligating groups, Cu_3_(HHTP)_2_ and Zn_3_(HHTP)_2_, a ∼2.5–2.7× increase in capacity was observed when switching from TEA^+^ to Li^+^ (Figure 3b). This was the largest increase for any systems studied and underscores the importance of oxygen-based functional groups in promoting charge storage with small, strongly interacting cations such as Li^+^. Co_3_(THT)_2_, featuring thiol (−S) ligating groups, also exhibited improved performance with Li^+^, though by a smaller factor (1.5×). In contrast, Ni_3_(HITP)_2_ and Cu_3_(HITP)_2_, which both contain protonated imino (−NH) ligating groups, showed no measurable improvement with Li^+^, despite differing metal nodes. These results demonstrate that Li^+^-driven enhancements in capacitive performance are primarily dictated by the identity of the ligating group and are independent of the metal node. These results are consistent with electric double-layer charge storage involving specific adsorption of partially desolvated cations at functional groups on the pore surface, rather than a redox-like or pseudocapacitive mechanism. While a small structural contribution due to the reduced interlayer spacing in HHTP-based MOFs, potentially enhancing local confinement and strengthening electrode–electrolyte interactions, cannot be ruled out, the lack of stacking differences between Co_3_(THT)_2_ and HITP-based MOFs, despite a clear disparity in Li^+^ capacity enhancement, supports the conclusion that chemical functionality dominates the observed variation.

Altogether, these results reveal that only MOFs with deprotonated hydroxy or thiol ligating groups show enhanced capacities with Li^+^ electrolytes, suggesting that protonated M–NH groups block the favorable interactions required for improved charge storage. The stronger enhancement in hydroxy-functionalized MOFs compared to thiol-functionalized analogues further underscores the role of charge-dense oxygen functionalities, which likely form stronger electrostatic interactions with Li^+^. These results highlight the critical influence of local pore chemistry on electric double-layer formation and motivate further mechanistic studies into ion-binding environments to better understand the molecular origins of the observed capacitive enhancements.

### Mechanistic Insights

To investigate why layered MOFs with hydroxy ligating groups exhibit the greatest improvement in capacitive performance with Li^+^ electrolytes, initial multiscale quantum mechanics/molecular mechanics (QM/MM) simulations were carried out to examine cation localization in negatively charged Cu_3_(HHTP)_2_ with Li^+^ and TEA^+^ electrolytes (SI Figure S23). These simulations reveal a strong electrostatic interaction between Li^+^ and the deprotonated oxygen atoms of the HHTP ligands, leading to a distinct population of Li^+^ ions located close to the pore walls of the charged MOF. In contrast, no such population is observed for TEA^+^, which remains more diffusely distributed within the pore.^50^ The residence time of the coordinated Li^+^ ions was measured at 0.3–1.0 ns, and their diffusion coefficient is approximately three times higher than that of TEA^+^ ions within the pores, likely due to their smaller size (SI Figures S24, S25). Further analysis reveals that Li^+^ exists in two primary coordination states, with this localization driven by the local electric field generated at the Cu–O_4_ moiety (SI Figures S26, S27). These results suggest specific Li^+^ binding to the functionalized pore surfaces and motivated further experimental investigation of the local cation environments.

Solid-state *ex situ* ^7^Li NMR spectroscopy was used to examine the Li^+^ adsorption environments in layered MOFs. Solid-state ^7^Li NMR spectra for uncharged Cu_3_(HHTP)_2_ and Zn_3_(HHTP)_2_ electrodes show two main environments: an ex-pore peak at approximately −3 ppm, corresponding to electrolyte outside the MOF pores, and an in-pore peak at approximately 4 ppm in Cu_3_(HHTP)_2_ and −1 ppm in Zn_3_(HHTP)_2_ (SI Figures S28, S29), consistent with previous work.^51^ Excitingly, Cu_3_(HHTP)_2_ exhibits a new signal at approximately −34 ppm upon negative charging which is outside the diamagnetic shift range of ^7^Li (Figure 4a; SI Figures S30, S31; SI Table S5) and suggests the formation of new paramagnetic Li^+^ environment.^52^ This negatively shifted peak is absent in Zn_3_(HHTP)_2_ (SI Figure S32), suggesting that the new Li^+^ environment observed in Cu_3_(HHTP)_2_ arises from interactions with paramagnetic Cu^2+^ nodes in the MOF, which are not present in the diamagnetic Zn-based analogue.

**Figure 4:**
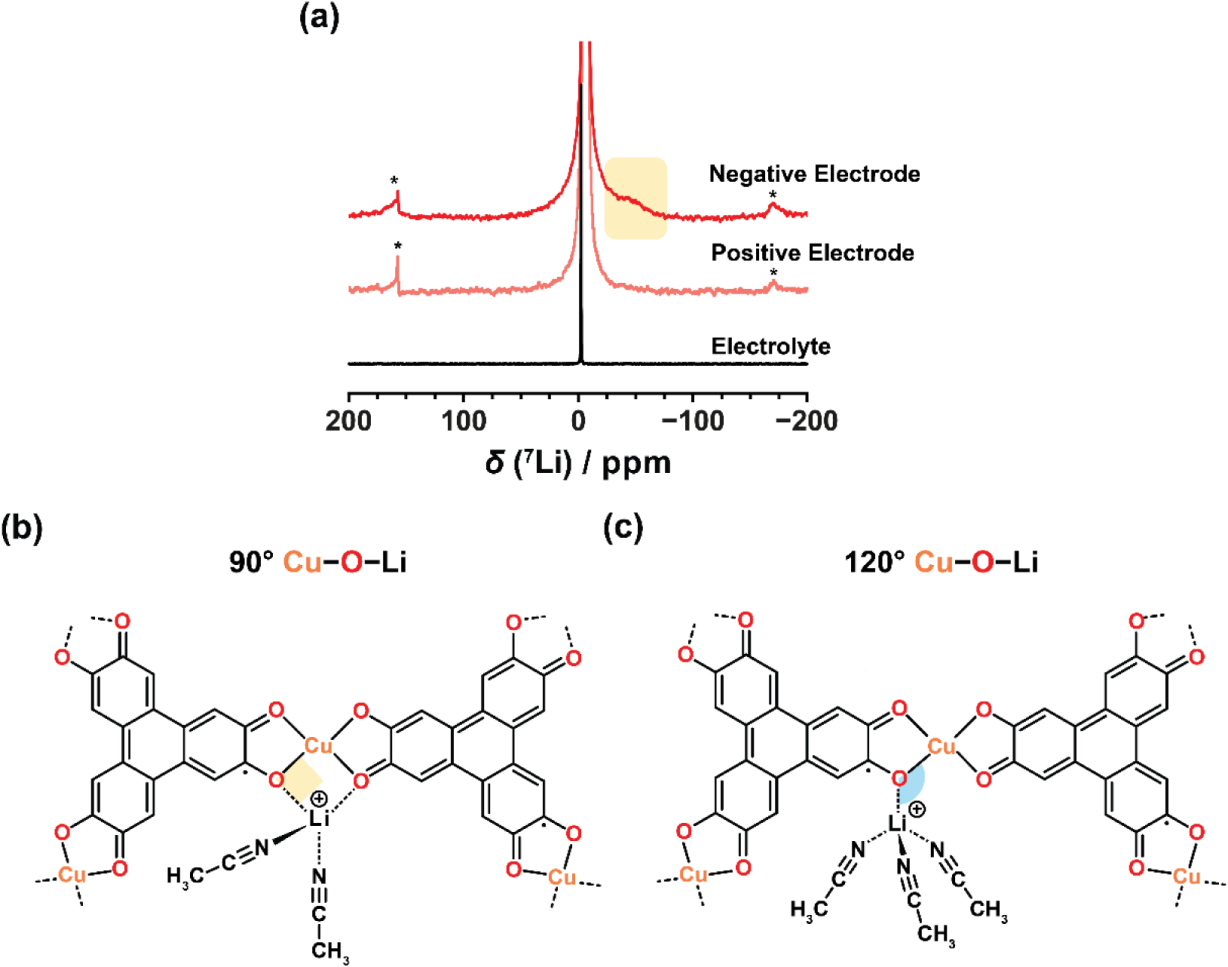
(a) ^7^Li solid-state *ex situ* NMR (9.4 T; 25 kHz magic-angle spinning) experiments of charged Cu_3_(HHTP)_2_ electrodes extracted from a symmetric two-electrode supercapacitor assembled with 1 M LiTFSI in acetonitrile. Spectra are normalized to the mass of MOF in each sample. The black spectrum (bottom) corresponds to the neat electrolyte. Asterisks denote spinning sidebands. (b) and (c) Simulated geometries of Li^+^ coordinated to a Cu_3_(HHTP)_2_ fragment: (b) Cu–O–Li in an approximately 90° configuration, with Li^+^ coordinated to two oxygen groups, and (c) Cu–O–Li in an approximately 120° configuration, with Li^+^ coordinated to one oxygen group. Solvation numbers shown may not be representative and were selected to enable computational convergence.

A similar negatively shifted environment (−60 to −100 ppm) is observed in ^23^Na *ex situ* NMR spectra of Cu_3_(HHTP)_2_ with Na^+^ electrolytes, appearing in both charged and uncharged samples with a charge-dependent chemical shift (SI Figure S33). Its presence with both Li^+^ and Na^+^ supports its assignment to specific alkali metal cation interactions with Cu–O sites and links these interactions to the enhanced capacitive performance observed with these cations.

To further probe the paramagnetic nature of the ^7^Li environments in Cu_3_(HHTP)_2_, variable-temperature NMR measurements were performed. Both the previously assigned in-pore and negatively shifted peaks showed temperature-dependent chemical shift changes, moving closer to the diamagnetic region with increasing temperature (SI Figure S34), consistent with paramagnetic interactions.^53^ This indicates two distinct in-pore Li^+^ environments in Cu_3_(HHTP)_2_, each associated with a different spin transfer mechanism involving the Cu^2+^ nodes. Building on the exploratory QM/MM simulations, paramagnetic NMR simulations were used to examine how local binding geometries influence the observed shifts, focusing on two representative coordination environments (Figure 4b, c). These geometries were chosen to reflect Li^+^–Cu–O bonding configurations relevant to the experimental system, rather than to reproduce full solvation shells. In the first geometry, Li^+^ adopts a square coordination across the O–Cu–O bonding unit, creating two ∼90 °Cu–O–Li pathways (91.6 °; Figure 4b). This site gives rise to a simulated Fermi contact shift of −34 ppm, closely matching the experimentally observed Δδ (chemical shift relative to the free electrolyte) of −31 ppm for the additional ^7^Li peak seen in negatively charged samples. This shift aligns with previous shifts for Li^+^ in ∼90° coordination with analogous M–O units, and suggests that the square coordination environment becomes more favorable upon negative charging (see Supporting Information for further discussion; SI Table S6).^54–56^ In contrast, the second geometry features a ∼120° (119.1°) Cu–O–Li pathway, with Li^+^ coordinated to a single oxygen atom in an overall tetrahedral coordination environment (Figure 4c). This site gives a small positive Fermi contact shift of +9 ppm, consistent with the Δδ of +7 ppm observed for the in-pore signal present in both charged and uncharged samples (SI Table S6).

Together, QM/MM and NMR simulations reveal that Li^+^ binds at functionalized pore edges in Cu_3_(HHTP)_2_, forming two distinct coordination environments with different coordination geometries and spin-transfer mechanisms. Notably, the square binding geometry site appears only in negatively charged samples, highlighting its role in the enhanced capacitive performance. Overall, the presence of these adsorption sites in Cu_3_(HHTP)_2_, and likely also in Zn_3_(HHTP)_2_ but not visibile in NMR experiments, emphasizes the strong cation binding affinity of hydroxy-functionalized MOFs.

To further investigate ion-binding sites, the local Li^+^ environments in Cu_3_(HITP)_2_ were probed using *ex situ* ^7^Li SSNMR spectroscopy. Unlike Cu_3_(HHTP)_2_, Cu_3_(HITP)_2_ shows no capacity enhancement with Li^+^ electrolytes (Figure 3b) but contains the same paramagnetic Cu^2+^ metal nodes. As expected, no new features were seen in the ^7^Li NMR spectra of positively or negatively charged Cu_3_(HITP)_2_, with no negatively shifted environments characteristic of paramagnetic coordination observed (SI Figure S35). The absence of this signal suggests that Li^+^ does not adopt the same square coordination geometry with Cu–NH units as it does with Cu–O units, supporting the conclusion that the specific Li^+^ binding site seen in Cu_3_(HHTP)_2_ is absent in Cu_3_(HITP)_2_. This reinforces the link between strong Li^+^ coordination and enhanced capacitive performance. A quantitative comparison of Li^+^ interaction energies for different ligand environments could provide further insight into these trends and represents an interesting direction for future theoretical investigation.

Electrochemical quartz crystal microbalance (EQCM) measurements were conducted to investigate the charge storage mechanism through a different approach. The mass change–charge (Δ*m*–*Q*) response supports a reversible, cation-dominated charging mechanism, with minimal anion involvement (SI Figure S36). This is consistent with previous findings for TEA^+^ electrolytes.^57^ The equivalent molecular weight change of ∼63 g mol^–1^ indicates that adsorption of each Li^+^ ion occurs with approximately 1–2 acetonitrile molecules (average of 1.4) during negative charging, lower than the typical solvation number of Li^+^ in acetonitrile (4–6).^58,59^ While the solvation numbers calculated from these measurements are only approximate, as they depend on assumptions regarding charging stoichiometry and minimal anion participation, the estimated reduced solvation compared to the bulk electrolyte indicates that Li^+^ ions lose part of their solvation shell upon adsorption in the MOF pores. This would lead to a decrease in solvent screening of the ionic charge, allowing for stronger interactions between Li^+^ and the oxygen-containing pore surface. This supports the earlier hypothesis that partial Li^+^ desolvation is required to enable the specific interactions with Cu–O sites that lead to the enhanced capacity.

While EQCM, NMR, and QM/MM results point to a cation-dominated charge storage mechanism involving specific interactions between Li^+^ ions and Cu–O functional groups in Cu_3_(HHTP)_2_, it is also important to consider whether Li^+^ intercalation between MOF layers could also contribute to the enhanced capacities. Indeed, previous studies with nonporous layered MOFs have attributed capacitance improvements with Li^+^ electrolytes to possible intercalation processes.^30,31^ To determine whether Li^+^ intercalation contributes to the enhanced capacity observed in Cu_3_(HHTP)_2_, operando wide-angle X-ray scattering (WAXS) experiments were performed with selective probing of the working electrode during electrochemical cycling. The interlayer spacing remained effectively constant throughout charge–discharge cycles, with a maximum variation of less than 0.1% (SI Figure S37). This negligible change is comparable to surface-adsorption-induced deformation and far below the ∼10% lattice expansion typically associated with Li^+^ intercalation into graphite electrodes (3.35 Å to 3.70 Å).^60^ These results provide strong evidence that Li^+^ does not intercalate into Cu_3_(HHTP)_2_ during charging. Complementary operando XRD measurements on symmetric cells showed similarly stable interlayer spacings and no shift or broadening of the (001) diffraction peak (SI Figures S38–S43). These results rule out intercalation as a contributing mechanism to the enhanced capacities seen for hydroxy-functionalized layered MOFs with alkali metal electrolytes. These experiments also show that the long-range crystal structure of Cu_3_(HHTP)_2_ is preserved over prolonged cycling, including voltage holds near the upper limit of the MOF stability window, as no significant changes in the diffraction peaks of the framework are observed. This indicates that the MOF structure remains intact during electrochemical cycling within the investigated voltage window.

Altogether, the results of mechanistic studies are most consistent with a mechanism in which partially desolvated Li^+^ ions interact with specific oxygen-containing Cu–O sites within the MOF pores, although additional factors such as changes in solvation structure, ion mobility, or the local dielectric environment may also contribute to the observed capacitive behavior. These interactions likely reduce the effective double-layer separation and enhance charge screening by δ^–^ hydroxy groups, enabling greater in-pore accumulation of cations at a given potential. This mechanism explains both the strong performance of hydroxy-functionalized MOFs and the moderate enhancement observed in thiol analogues, with the lower δ^–^ charge density of the M–S units result in weaker interactions and lower charge screening. MOFs with imino (M–NH) ligands show no enhancement likely due to steric blocking of favorable adsorption geometries, as supported by NMR. These results highlight the dominant role of pore chemistry in modulating electrode–electrolyte interactions and further rationalize the trend across alkali metal cations: as the strength of ion–surface interactions decreases across the alkali-metal series (Li^+^ > Na^+^ > K^+^), double-layer thickness increases and capacitive performance decreases. The absence of performance enhancement with Mg^2+^ compared to TEA^+^, despite its higher ionic charge, suggests that partial desolvation is required to enable strong electrode–electrolyte interactions.

These findings align with previous simulations linking capacitance in porous carbons to ion-specific adsorption and local charge compensation.^61^ Recent experiments further show that structural disorder in carbons can enhance performance via an increased density of edge sites, which favor ion adsorption.^62^ The prevalence of functionalized pore edges in layered MOFs supports the interpretation that edge-site ion binding contributes significantly to the performance gains observed in hydroxy-functionalized systems. Together, these results provide molecular-level insight into how functional group chemistry influences ion adsorption and capacitive behavior in conductive MOF electrodes. Although the present conclusions are derived from triphenylene-based MOFs studied in acetonitrile electrolytes, they suggest that controlling pore functionality may be an effective strategy for tuning electrode–electrolyte interactions in related nanoporous materials.

## Conclusions

This study provides insight into how electrode–electrolyte interactions influence electric double-layer structure and electrochemical performance. Using layered MOFs as model systems with well-defined pore chemistries, we showed that both electrolyte cation identity and pore functionality play a key role in governing charge storage. An increase in the electrochemical capacity of Cu_3_(HHTP)_2_ was observed in 1 M TFSI^–^ electrolytes from TEA^+^ to Li^+^, following the trend TEA^+^ < K^+^ < Na^+^ < Li^+^. This behavior is consistent with partial cation desolvation within the MOF pores, enabling specific interactions with the oxygen-containing functional groups of Cu_3_(HHTP)_2_ that enhance charge screening and electric double-layer formation. Solid-state ^7^Li NMR spectroscopy revealed the presence of a binding site for Li^+^ in negatively charged Cu_3_(HHTP)_2_ electrodes, consistent with localized adsorption at Cu–O units. Operando X-ray scattering ruled out Li^+^ intercalation, supporting the conclusion that the observed capacity enhancements arise from ion adsorption at functionalized pore sites. Electrochemical measurements across a family of MOFs showed that performance enhancements correlate with ligating group identity: M–O bonding units yielded the highest capacity increase with Li^+^, followed by M–S bonding units, while M–NH groups showed no improvement due to inhibited Li^+^ binding. Together, these results provide molecular-level insights into how pore functionality and ion properties influence double-layer formation in conductive MOFs and highlight the role of electrode–electrolyte interactions in governing charge storage behavior in nanoporous electrodes.

## Methods

### Synthesis

Cu_3_(HHTP)_2_ was synthesized by modifying an existing literature procedure.^63^ A solution of Cu­(NO_3_)_2_·3H_2_O (0.127 g, 0.526 mmol, 1.65 equiv) and aqueous ammonia (35%, 0.883 mL, 50 equiv) in distilled water (2 mL) was prepared. The resulting royal blue solution was added dropwise to a dispersion of H_6_HHTP (0.103 g, 0.318 mmol, 1.00 equiv) in distilled water (8.4 mL). The resulting mixture was heated in a furnace oven at 80 °C for 24 h. The dark blue precipitate formed was separated by centrifugation. The precipitate was then washed successively with water (3 × 30 mL), ethanol (3 × 30 mL), and acetone (3 × 30 mL). Washing was performed by centrifuging the precipitate with the desired washing solvent for 15–30 min before removing the supernatant layer and replacing with fresh washing solvent. No soaking of the precipitate was performed. The precipitate was then filtered by vacuum filtration, and the resulting dark blue powder was dried at 80 °C under dynamic vacuum for 96 h on a Schlenk line before being stored in a N_2_-filled glovebox until used.

Ni_3_(HITP)_2_ was synthesized by modifying existing literature procedures.^48,64^ A solution of NiCl_2_·6H_2_O (323 mg, 1.36 mmol, 1.5 equiv) in distilled water (20 mL) was added to a solution of 2,3,6,7,10,11-hexaaminotriphenylene hexahydrochloride, HATP·6HCl (487 mg, 0.91 mmol, 1 equiv) in distilled water (140 mL). To this was added 4.5 mL of concentrated aqueous ammonia (35%, 890 equiv). The resulting mixture was heated in an oil bath at 60 °C for 2 h with air bubbling and stirring. The resulting black precipitate was separated from the reaction mixture by centrifugation and washed successively with water (8 × 135 mL) and ethanol (3 × 135 mL). Washing was performed by centrifuging the precipitate with the desired washing solvent for approximately 8 h before removing the supernatant layer and replacing with fresh washing solvent. The precipitate was then filtered by vacuum filtration, and the resulting black powder was dried at 90 °C under dynamic vacuum for 96 h on a Schlenk line before being stored in a N_2_-filled glovebox until used.

Zn_3_(HHTP)_2_ was synthesized by modifying an existing literature procedure.^46^ H_6_HHTP (111 mg, 0.342 mmol, 1 equiv) and zinc acetylacetonate (Zn­(C_5_H_7_O_2_)_2_; 159 mg, 0.587 mmol, 1.7 equiv) were added to distilled water (15 mL) and sonicated for 10 min until all solid was dissolved. Anhydrous N-methyl-2-pyrrolidone, NMP, (1.5 mL, 46 equiv) was added, and the resulting mixture sonicated for 30 min. The resulting mixture was heated in a furnace oven at 80 °C for 12 h. The dark blue precipitate formed was separated by centrifugation. The precipitate was then washed successively with water (3 × 30 mL), ethanol (3 × 30 mL), and acetone (3 × 30 mL). Washing was performed by centrifuging the precipitate with the desired washing solvent for 15–30 min before removing the supernatant layer and replacing with fresh washing solvent. No soaking of the precipitate was performed. The precipitate was then filtered by vacuum filtration, and the resulting dark blue powder was dried at 80 °C under dynamic vacuum for 96 h on a Schlenk line before being stored in a N_2_-filled glovebox until used.

Co_3_(THT)_2_ was synthesized by modifying existing literature procedures.^47,65^ Under strictly anaerobic conditions, a 40 mL aqueous solution of CoCl_2_.6H_2_O (40 mg, 0.168 mmol) was prepared in a 120 mL jar. Separately, a suspension of triphenylene-2,3,6,7,10,11-hexathiol, THT (2.5 mg, 0.024 mmol) in NMP (1 mL) was prepared and then diluted with ethyl acetate until the total volume of the suspension reached 5 mL. This solution was sealed and briefly sonicated to form a uniform cloudy suspension. Ethyl acetate (35 mL) was gently layered on top of the aqueous Co^2+^ solution to create a liquid–liquid interface before the suspension of THT was gently added to the ethyl acetate layer. The resulting mixture was allowed to stand at room temperature. A black film appeared at the liquid–liquid interface over 5 days. The obtained films were isolated by decanting the water and ethyl acetate. Subsequently, solvent exchange was performed three times each with 20 mL water and 20 mL methanol, and films were allowed to evaporate to dryness. Multiple syntheses were performed to produce enough material for electrochemical measurements. The resulting black powder was dried at room temperature under dynamic vacuum for 24 h, before being further dried at 75 °C for 72 h under dynamic vacuum. The material was then stored in a N_2_-filled glovebox until used.

Cu_3_(HITP)_2_ was synthesized by modifying an existing literature procedure.^66^ A suspension of CuSO_4_·5H_2_O (7 mg, 0.014 mmol, 1.5 equiv) was suspended in dimethylacetamide, DMA, (1.5 mL) and sonicated for 10 min to form a light green solution. Then, a solution of HATP·6HCl (10 mg, 0.0093 mmol, 1 equiv) in distilled water (3 mL) was added and, and the resulting mixture was sonicated for 5 min to ensure homogeneity. To this was added 4 mL of aqueous sodium acetate solution (2 M; 860 equiv). The mixture was then heated in an oil bath at 65 °C for 2 h with stirring and while being open to air. Multiple syntheses were performed to produce enough material for electrochemical measurements, and 10 batches were combined and washed together. The resulting black precipitate was separated from the reaction mixture by centrifugation and washed successively with water (3 × 50 mL), methanol (3 × 50 mL), and acetone (3 × 50 mL). Washing was performed as described above for Zn_3_(HHTP)_2_. The precipitate was then filtered by vacuum filtration, and the resulting black powder was dried at 75 °C under dynamic vacuum for 96 h in a vacuum oven before being stored in a N_2_-filled glovebox until used.

### Material Characterization

Laboratory powder X-ray diffraction (XRD) data were collected on a Malvern Panalytical Empyrean instrument, equipped with an X’celerator Scientific detector using nonmonochromated Cu K_α_ radiation (λ = 1.5418 Å). Samples were placed in a glass sample holder and measured in reflection geometry with sample spinning. The data were collected at room temperature over a 2θ range of 3–50°, with an effective step size of 0.017° and a total collection time of 1 h. Powder XRD data were collected for all layered MOFs synthesized in this work.

Low-pressure N_2_ isotherms (adsorption and desorption) were collected using an Anton Paar Autosorb iQ-XR at 77 K. *Ex situ* degassing (90 °C, 24 h) was performed and isotherms were collected over 24–36 h. Sorption isotherms were evaluated in AsiQwin version 5.21 software. Material BET areas were calculated from isotherms using the BET equation and Rouquerol’s consistency criteria implemented in AsiQwin. Pore size distribution fittings were conducted in AsiQwin using N_2_ at 77 K on carbon (cylindrical pores) QSDFT model with a bin pore width of 0.5 Å. Gas sorption data were collected for powder samples of all layered MOFs synthesized in this work.

### Electrode Film Preparation

Freestanding electrode films were prepared by modifying an existing literature procedure.^27^ In brief, the electrode material was ground along with 10 wt % acetylene black, added as a conductive additive (measured BET area = 62 m^2^ g^–1^), in a vial before ethanol (approximately 1.5 mL) was added to produce a loose slurry. This was sonicated for 15 min before being added to PTFE dispersion (60 wt % in water) in a few drops of ethanol. The slurry was stirred by hand for approximately 20 min under ambient conditions. The film was formed upon drying of the slurry and was then kneaded for approximately 20 min to ensure homogeneity before being rolled into a freestanding electrode film using a homemade aluminum rolling pin. The film was dried at 75 °C under dynamic vacuum for at least 48 h to remove remaining ethanol.

The masses of components were calculated so that the final layered MOF electrode films had a composition of 85 wt % MOF, 10 wt % acetylene black, and 5 wt % PTFE. YP50F electrode films were made with 85 wt % YP50F, 10 wt % acetylene black, and 5 wt % PTFE, and were dried at 100 °C under dynamic vacuum. All films had a thickness of approximately 250 μm.

### Supercapacitor Assemblies

Symmetric two-electrode supercapacitor cells were assembled as coin cells, while three-electrode cells and supercapacitors for *ex situ* NMR spectroscopy experiments were assembled in Swagelok PFA-820-3 tee tube fittings.

For symmetric two-electrode supercapacitors, electrodes were cut from freestanding electrode films in a N_2_-filled glovebox with a diameter of 6.35 mm. For a given cell, the difference in mass between the two electrodes was ≤5%. 1 M solutions of TEATFSI, LiTFSI, NaTFSI, KTFSI, and Mg­(TFSI)_2_ in anhydrous acetonitrile were used as electrolytes. All electrolyte solutions were prepared in a N_2_-filled glovebox. The amount of electrolyte added was kept constant between cells (200 μL). Whatman glass microfiber filter (GF/A) was used as a separator, and two separators were added to each cell. Coin cells were sealed in the glovebox using a hydraulic crimper. Swagelok supercapacitors for *ex situ* NMR experiments were assembled with homemade stainless-steel plugs as current collectors and sealed hermetically by hand. All supercapacitors were removed from the glovebox for electrochemical testing.

For three-electrode cells, working electrodes were cut from freestanding electrode films in a N_2_-filled glovebox with a diameter of 4.76 mm. Overcapacitive YP80F activated carbon film electrodes with an areal mass loading of 35–40 mg cm^–2^ were used as counter electrodes, giving a mass ratio between the working and counter electrode of approximately 1:3. Ag wire was used as a pseudoreference electrode. For supercapacitors for *ex situ* NMR spectroscopy experiments, all electrodes were cut with a diameter of 6.35 mm. The amount of electrolyte added to all cells was kept constant (750 μL). Whatman glass microfiber filter (GF/A) was used as a separator, and two separators were added to each cell. The cells were hermetically sealed by hand and removed from the glovebox for testing. All potentials discussed for the three-electrode cell are referenced to Ag.

Electrochemical cells for use in operando XRD measurements were assembled in custom-made AMPIX electrochemical cells as symmetric two-electrode supercapacitors.^67^ Electrodes were cut from freestanding Cu_3_(HHTP)_2_ electrode films in a N_2_-filled glovebox with a diameter of 9.53 mm. For a given cell, the difference in mass between the two electrodes was ≤5%. Cu_3_(HHTP)_2_ electrodes were placed onto circular Al foil current collectors (diameter 11.11 mm) prior to assembling the cell. 1 M solutions of TEATFSI and LiTFSI in anhydrous acetonitrile were used as electrolytes. The amount of electrolyte added was kept constant between cells (350 μL). Whatman glass microfiber filter (GF/A) was used as a separator, and two separators were added to each cell. Separators were cut with a diameter of 14.29 mm. No O-rings were used in the AMPIX cells. This was required to ensure sufficient pressure and low cell resistances. All cells were assembled in an Ar-filled glovebox at Diamond Light Source and were hermetically sealed with screws. *In situ* XRD data were only collected for Cu_3_(HHTP)_2_ with both TEATFSI and LiTFSI electrolytes.

Symmetric two-electrode supercapacitor cells were assembled with 1 M TEATFSI and LiTFSI in acetonitrile electrolytes for all layered MOF studied in this work, as well as for YP50F porous carbon. Symmetric two-electrode supercapacitor cells with 1 M NaTFSI, KTFSI, and Mg­(TFSI)_2_ in acetonitrile electrolytes were only assembled with Cu_3_(HHTP)_2_. Three-electrode cells were assembled with Cu_3_(HHTP)_2_ as the working electrode material and 1 M LiTFSI in acetonitrile as the electrolyte. Supercapacitors for *ex situ* NMR spectroscopy were assembled with 1 M LiTFSI in acetonitrile electrolyte with Cu_3_(HHTP)_2_, Zn_3_(HHTP)_2_, and Cu_3_(HITP)_2_, and with 1 M NaTFSI in acetonitrile electrolyte with Cu_3_(HHTP)_2_.

### Electrochemical Measurements

All electrochemical measurements on two-electrode coin cells and Swagelok cells were carried out using Biologic VSP-3e and SP-150 potentiostats, and a Biologic BCS-800 series ultraprecision battery cycler.

All experimental capacity values were calculated after removing the contributions of acetylene black and PTFE that are also present in the electrodes. For two-electrode experiments, specific capacity values are normalized by the average mass of electroactive material in a single electrode (i.e., a pseudo single electrode measurement independent of device architecture). Current densities were calculated by dividing the current applied during the GCD experiment, *I*, by the average mass of active material per electrode, *m*. Capacity is reported as the primary energy storage performance metric, in line with previous recommendations for data reporting.

EIS measurements were performed in the frequency range from 1 MHz to 3 mHz (decreasing frequency) at the open circuit voltage (OCV) using a single sinusoidal signal with a sinus amplitude of 10 mV and drift correction applied.

The electrochemical stability window for each MOF–electrolyte system was determined experimentally from CV measurements performed at progressively increasing final cell voltages. The stable potential window was defined as the voltage range over which the current response remained predominantly capacitive (i.e., quasi-rectangular) and free from evidence of irreversible electrochemical processes.

### QM/MM Simulations

The localization of Li^+^ and TEA^+^ cations within the pores of Cu_3_(HHTP)_2_ was investigated using QM/MM simulations. The iterative DFT-CES (Density Functional Theory in Classical Explicit Solvents) method was employed, coupling Quantum ESPRESSO (version 7.2)^68,69^ for the quantum mechanical treatment of the negatively charged MOF layers (PBE + D3 + U) with LAMMPS^70^ for the classical description of the electrolyte, using the OPLS-AA force field. The full computational setup, including system construction and convergence parameters, follows that reported previously.^50^ The nonbonded interaction parameters for Li^+^ were taken from a previous study.^71^

### Solid-State NMR Measurements

Solid-state NMR experiments were performed on a Bruker Avance Neo spectrometer with a magnetic field strength of 9.4 T, corresponding to a ^1^H Larmor frequency of 400.2 MHz, using a Bruker 2.5 mm double-resonance MAS probe. All spectra were recorded under magic angle spinning of 25 kHz, and the 90° pulse length was optimized for every sample. ^7^Li chemical shifts were referenced externally to lithium chloride at −1.0 ppm. The temperature was calibrated in variable temperature NMR experiments using the temperature-dependent *T*_1_(^79^Br) of KBr, measured using an inversion–recovery pulse sequence.^72^

*Ex situ* NMR spectroscopy experiments were performed on electrodes extracted from disassembled Swagelok supercapacitors assembled as described above. The cells were precycled by running cyclic voltammograms up to (i) 0.5 V at a scan rate of 5 mV s^–1^ for 30 cycles for Cu_3_(HHTP)_2_ and Cu_3_(HITP)_2_; (ii) 1.0 V at a scan rate of 5 mV s^–1^ for 30 cycles for Zn_3_(HHTP)_2_. All cells were then left for a minimum of 12 h to allow for complete wetting of the electrodes. For charged samples, the cells were then held at a constant cell voltage for 1 h before being immediately returned to the glovebox for disassembly. All cells were initially held at the open circuit voltage for 0.5 h. Cells assembled with Cu_3_(HHTP)_2_ and Cu_3_(HITP)_2_ were then held at a cell voltage of 0.5 V while cells assembled with Zn_3_(HHTP)_2_ were held at a cell voltage of 1.0 V. For uncharged samples, no constant voltage was applied following precycling. Electrodes were packed into zirconia MAS rotors (2.5 mm outer diameter) in a N_2_-filled glovebox. The rotor was weighed before and after adding the MOF material. During supercapacitor disassembly, the two electrodes were packed simultaneously by two independent researchers into separate rotors to minimize acetonitrile evaporation.

All spectra are normalized according to the number of scans performed. Spectra presented in the same figure are further normalized to the mass of MOF in the sample. All spectra were fitted using DMfit software using a CSA model (Haeberlen convention) to fit spinning sidebands.^73^

### Paramagnetic NMR Simulations

Spin polarized solid-state hybrid density functional theory (DFT) calculations were performed in the CRYSTAL17 code^74^ using the PBE0 hybrid functional.^75^ A total energy convergence of 2.72 × 10^–6^ eV was used for all calculations, with a Monkhorst–Pack k-point mesh of 5 × 5 × 1 and integral tolerances of 10^–7^, 10^–7^, 10^–7^, 10^–7^, and 10^–14^, as defined in the CRYSTAL17 manual.

The structure of Cu_3_(HHTP)_2_ was modeled as a single Cu_3_(HHTP)_2_ layer within the *ab* plane of a periodic unit cell. ∼12 Å of vacuum was included along the *c*-axis to separate the layer from its neighboring image. The atomic positions and unit cell parameters were then optimized to find a low energy configuration for further paramagnetic NMR calculations. Root-mean-square convergence criteria 8.16 × 10^–3^ and 3.27 × 10^–2^ eV were used for maximum gradients and displacements, respectively, during the geometry optimization. A single Li^+^ ion was then introduced into the Cu_3_(HHTP)_2_ cell adjacent to the Cu node, coordinated by 2 or 3 ACN molecules. A charge neutralizing background was applied to the calculations containing Li^+^ ions to compensate for the additional positive charge. The atomic positions of all atoms were allowed to optimize under fixed cell conditions. All-electron POB-triple-ζ valence + polarization (POB-TZVP-REV2) basis sets were taken from the CRYSTAL online repository and used for Cu, C, O, H, N and Li without further modification.^76^

^7^Li Fermi contact shifts were calculated from hybrid DFT calculations of the hyperfine coupling constant, *A*_iso_, at 0 K using the approach developed in previous studies.^77,78^ *A*_iso_ calculated at 0 K were scaled to finite temperature using a scaling factor, Φ:

$$\Phi =\frac{B_{0}\mu_{\mathrm{eff}}^{2}}{3k_{B}g_{e}\mu_{B}S_{\mathrm{form}}\left(T-\theta \right)}$$

where *B*_0_ is the external magnetic field, *k*_B_ is Boltzmann’s constant, μ_B_ is the Bohr magneton and *g*_e_ is the free electron g factor. *T* is experimental temperature, which was assumed to be 320 K due to frictional heating from magic-angle spinning. *S*_form_ is the formal spin angular momentum, which is assumed to be *S*_form_ = 1/2 for Cu^2+^ and HHTP^3–^. θ and μ_eff_ are the Weiss constant and effective magnetic moment, respectively. The values of θ and μ_eff_ were approximated as 0 K and 1.73 μ_B_, respectively, equivalent to the Curie spin approximation for a *S* = 1/2 system. These values agree with magnetic measurements of the Cu_3_(HHTP)_2_ system.^79^

To isolate the contribution from the unpaired electrons on the Cu^2+^ node, $\left(\delta_{\mathrm{node}}^{\mathrm{7Li}}\right)$ , the spin flipping approach was used in which the Fermi contact shift was initially calculated in the ferromagnetic state $(\delta_{\mathrm{Ferro}}^{\mathrm{7Li}}$ ) and then the spin on the Cu node adjacent to the alkali ion was flipped $(\delta_{\mathrm{Flipped}}^{\mathrm{7Li}}$ ). The difference in the shift gives the Fermi contact contribution from the node: $\delta_{\mathrm{node}}^{\mathrm{7Li}}=(\delta_{\mathrm{Ferro}}^{\mathrm{7Li}}-\delta_{\mathrm{Flipped}}^{\mathrm{7Li}})/2$ .^75^

The Fermi contact interaction on ^7^Li sites from the Cu^2+^ (*S* = 1/2) node was calculated for different solvent configurations (2 acetonitrile molecules and 3 acetonitrile molecules).

### Electrochemical Quartz Crystal Microbalance Measurements

EQCM measurements were performed with an AT-cut Au-coated quartz crystal with an oscillating frequency of 9 MHz. A slurry containing 85 wt % Cu_3_(HHTP)_2_ powder, 10 wt % acetylene black, and 5 wt % polyvinylidene fluoride (PVDF) binder in *N*-methyl-2-pyrrolidone (NMP) was spray-coated onto the Au-coated surface of the quartz crystal. The sample-coated quartz crystal was dried at 80 °C under dynamic vacuum for 24 h to remove remaining NMP and then used as the working electrode in EQCM cells. Platinum wire was used as the counter electrode, and Ag wire was used as a pseudoreference electrode. A 1 M solution of LiTFSI in anhydrous acetonitrile was used as the electrolyte. EQCM cells were assembled in a N_2_-filled glovebox. EQCM electrochemical measurements were carried out using a Metrohm Autolab electrochemical workstation and a rotating QCM system in tandem to allow for simultaneous recording of frequency and electrochemistry data. EQCM measurements were only performed on Cu_3_(HHTP)_2_ with 1 M LiTFSI in anhydrous ACN electrolyte.

### Operando X-ray Diffraction and Scattering Measurements

Operando synchrotron X-ray diffraction measurements were performed at the I11 beamline at Diamond Light Source. All electrochemical measurements were carried out on a Neware BTS-4000 battery tester. Before starting operando XRD measurements, each cell was precycled by running cyclic voltammograms up to 0.5 V at a scan rate of 5 mV s^–1^ for 20 cycles. This helped to stabilize the electrochemical response of the cell. Operando diffraction patterns were collected under ambient conditions using a Mythen II position-sensitive detector (PSD). The wavelength and intrinsic peak-shape parameters were refined against a known Si 640c NIST standard. The refined wavelength was 0.493463 Å (∼25 keV), and the beam size was approximately 1.5 mm × 0.5 mm. Multiple cells were assembled on a motorized stage and translated into the X-ray beam periodically to increase the efficiency of the XRD measurements. Both electrodes were irradiated during operando XRD measurements.

All of the supercapacitors used for the operando XRD experiments were cycled using a constant-current/constant-voltage (CC/CV) charging protocol. The cells were first charged up to a cell voltage of 0.5 V using a constant-current experiment at a current density of 0.1 A g^–1^. The cell voltage was then held at 0.5 V for 3 min before the cell was discharged, again with a constant-current experiment at a current density of 0.1 A g^–1^. This procedure was then repeated two further times to give three cycles in total. During electrochemical cycling, XRD data were collected on a given cell approximately every 93 s. During each scan, data were collected over a 2θ range of 1–90 ° with a step size of 0.004°, with each scan taking approximately 20 s. For a given sample, a total of 20 separate measurements were taken, covering a range of different charging states. Timings were chosen to ensure that at least three separate XRD scans were performed on each cell while it was held at the maximum cell voltage of 0.5 V. A background XRD pattern of each cell was taken prior to electrochemical cycling.

Analysis and visualization of operando XRD data was performed using a custom Bokeh Python code.

Operando Small and Wide Angle X-ray Scattering (SAXS/WAXS) experiments were performed at the Austrian SAXS beamline at the ELETTRA Sincrotrone Trieste. Two circular electrodes, each approximately 6.3 mm in diameter and 250 μm in thickness, were punched from a free-standing Cu_3_(HHTP)_2_ film. One of the electrodes featured a central cutout measuring 2.5 × 1 mm, enabling selective irradiation of a single electrode during measurement. The electrodes were stacked in a conventional two-electrode sandwich configuration using platinum foil current collectors, Whatman glass microfiber filter (GF/A) separator, and 0.5 M LiTFSI in propylene carbonate electrolyte. A custom-made operando electrochemical cell was used,^80^ and the assembly was carried out inside an argon-filled glovebox. Following initial measurements, the cell was briefly opened to air (∼30 s) to inspect and realign the electrodes following observation of a weak signal. The operando measurements presented were performed after this brief exposure to air.

Cyclic voltammetry was carried out using a Gamry Interface 1010B potentiostat at a scan rate of 0.5 mV s^–1^ within a voltage window of 0.6 V. The synchrotron beam had a photon energy of 16.0 keV and was focused to a spot size of 2 × 0.5 mm at the position of the counter electrode cutout. SAXS/WAXS data were recorded using Pilatus3 1M and Pilatus3 100k 2D detectors, respectively, with an exposure time of 10 s and a wait time of 11 s, resulting in one acquisition every 21 s.

All two-dimensional scattering patterns were azimuthally integrated to obtain one-dimensional scattering profiles, representing intensity as a function of the scattering vector magnitude, (*q* = (4π sin θ)/λ), where 2θ is the scattering angle and λ the photon wavelength. Standard data normalization and correction procedures were applied as per beamline protocols, including corrections for fluctuations in primary beam intensity.

The layer spacing, *c*, was then calculated from the position of the (001) stacking peak following:

$$c=\frac{2\pi }{q_{001}}$$

Peak fitting was performed using a Pseudo-Voigt peak shape and a decaying exponential background.

## Supplementary Material



## Data Availability

All raw experimental data files are available in the Cambridge Research Repository, Apollo: 10.17863/CAM.119589.

## Notes

Sample characterization data, additional electrochemical analysis, supplementary NMR measurements, details of paramagnetic NMR and QM/MM simulations, EQCM data, and additional operando X-ray measurements (PDF)
