# Supplementary material for: Controlling Electrode–Electrolyte Interactions to Enhance Capacitance
Source: J Am Chem Soc. 2026 Apr 13;148(15):15694–706. doi: 10.1021/jacs.5c20988 (PMC13107464; doi:10.1021/jacs.5c20988)
Supplement: Supplementary file 1 [file ja5c20988_si_001.pdf]

Supplementary Information For:

Controlling Electrode–Electrolyte Interactions to Enhance Capacitance

Jamie W. Gittins[1†], Chloe J. Balhatchet[1†], James Hill[1,2], Teedhat Trisukhon[1], Malina Seyffertitz[1,3], Seung-Jae Shin[4], Yashna Khakre[5], Kangkang Ge[6], Thomas Kress[1], Smaranda C. Marinescu[5], Aron Walsh[7], Oskar Paris[3], Ieuan D. Seymour[8], Alexander C. Forse[1*]

[1] Yusuf Hamied Department of Chemistry, University of Cambridge, Lensfield Road, Cambridge CB2 1EW, UK.

[2] Wolfson Catalysis Centre, Department of Chemistry, University of Oxford, South Parks Road, Oxford, OX1 3QR, UK.

[3] Department Physics, Mechanics and Electrical Engineering, Montanuniversität Leoben, Franz-Josef-Straße 18, Leoben 8700, Austria.

[4] School of Energy and Chemical Engineering, Ulsan National Institute of Science and Technology (UNIST), Ulsan 44919, Republic of Korea.

[5] Department of Chemistry, University of Southern California, California, Los Angeles 90089, USA.

[6] CIRIMAT, UMR CNRS 5085, Université de Toulouse, Toulouse 31062, France.

[7] Thomas Young Centre & Department of Materials, Imperial College London, London SW7 2AZ, UK.

[8] Advanced Centre for Energy and Sustainability (ACES), Department of Chemistry, University of Aberdeen, Meston Walk, Aberdeen, AB24 3UE, UK.

[†] Authors contributed equally.

[*]Corresponding author’s email: acf50@cam.ac.uk

Contents

Figure S1 4
Figure S2 5
Figure S3 6
Figure S4 7
Figure S5 8
Figure S6 10
Table S1 11
Figure S7 12
Figure S8 14
Figure S9 15
Figure S10 16
Figure S11 .... **Error! Bookmark not defined.**
Figure S12 .... **Error! Bookmark not defined.**
Figure S13 18
Figure S14 19
Table S2 20
Figure S15 21
Figure S16 22
Figure S17 23
Figure S18 24
Table S3 25
Figure S19 26
Figure S20 27
Table S4 28
Figure S21 30
Figure S22 35
Figure S23 36
Figure S24 37
Figure S25 38
Table S5 39
Figure S26 40
Figure S27 41
Figure S28 42
Table S6 43
Figure S29 44

Figure S30 45
Figure S31 46
Figure S32 47
Figure S33 48
Figure S34 49
Figure S35 50
Figure S36 51
Figure S37 52
Figure S38 53
Additional Discussion 54
Paramagnetic NMR Calculations 54
Figure S39 54
Fermi Contact Shifts 54
Bibliography 55

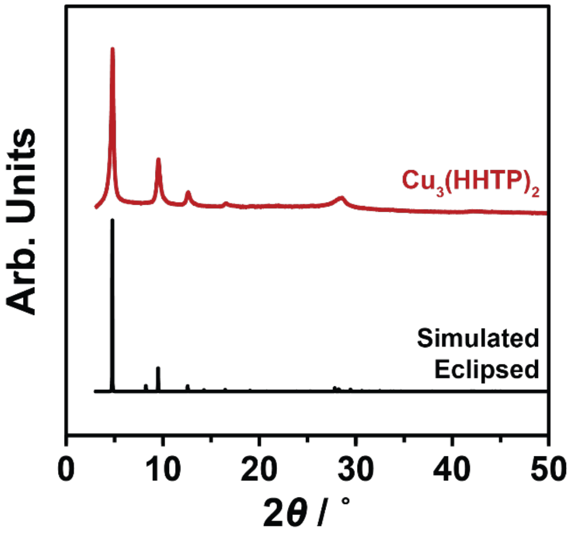

Figure S1: Experimental powder XRD pattern from $Cu_3(HHTP)_2$ synthesised in this work (dark red; Sample 1), compared to the simulated XRD pattern for a layered triphenylene MOF with eclipsed layer stacking (black).

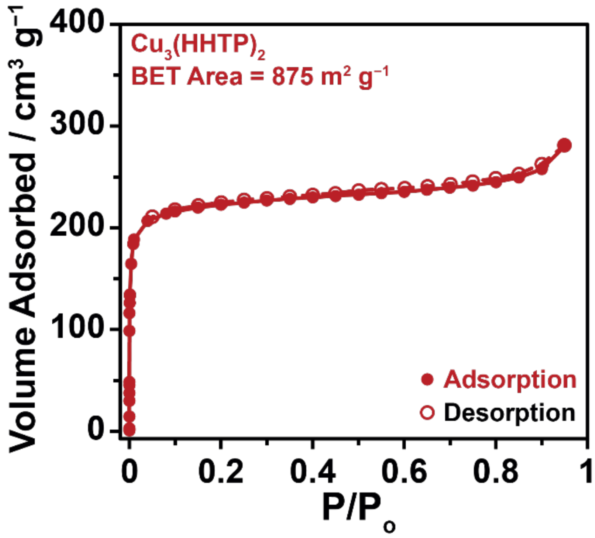

Figure S2: 77 K N_2 sorption isotherm of $Cu_3(HHTP)_2$ synthesised in this work (Sample 1). Adsorption data are shown with filled circles, while desorption data are shown with unfilled circles. From this, a BET surface area of 875 m^2 g^{-1} was determined.

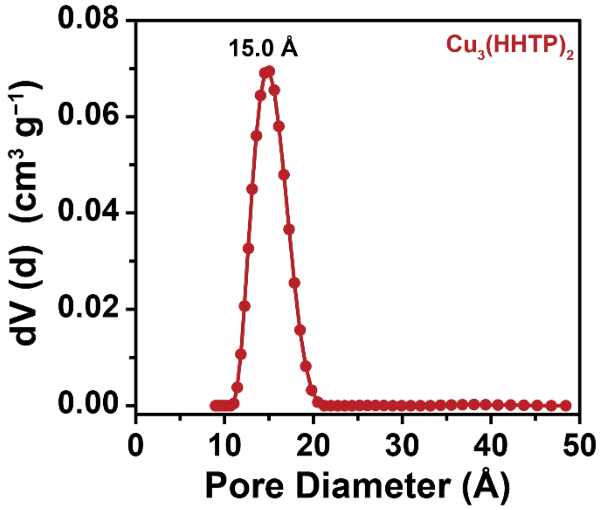

Figure S3: Pore size distribution for $Cu_3(HHTP)_2$ synthesised in this work (Sample 1), calculated using a N_2 at 77 K on carbon (cylindrical pores) quenched solid density functional theory (QSDFT) model from the corresponding N_2 isotherm.

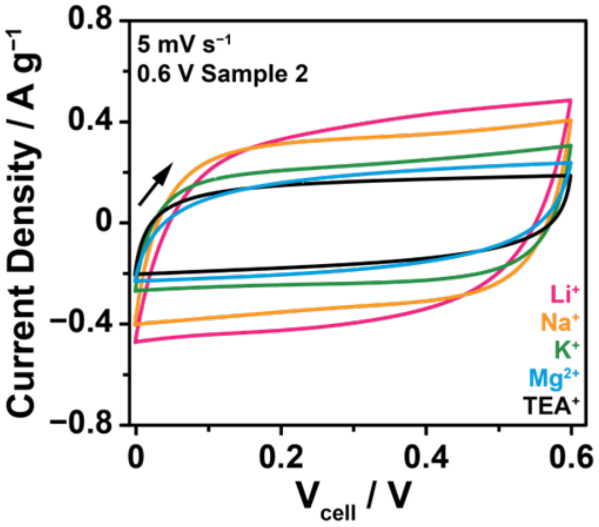

Figure S4: Repeat CVs obtained at a scan rate of 5 mV s^{-1} up to 0.6 V from two-electrode symmetric supercapacitors assembled with $Cu_3(HHTP)_2$ electrodes (Sample 2) and 1 M solutions of LiTFSI, NaTFSI, KTFSI, TEATFSI, and $Mg(TFSI)_2$ in acetonitrile electrolytes. This data was acquired on a different sample of $Cu_3(HHTP)_2$ than the CV data presented in Figure 1 of the Main Text and confirms the reproducibility of the results. The black arrow indicates the scan direction.

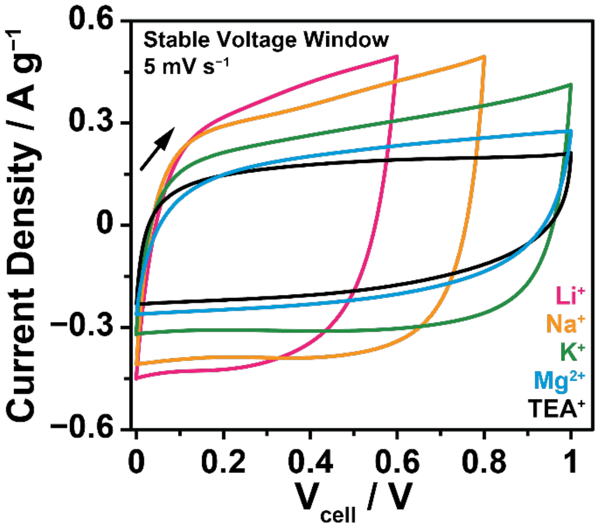

Figure S5: CVs obtained at a scan rate of 5 mV s^{-1} from two-electrode symmetric supercapacitors assembled with $Cu_3(HHTP)_2$ electrodes (Sample 1) and 1 M solutions of LiTFSI, NaTFSI, KTFSI, TEATFSI, and $Mg(TFSI)_2$ in acetonitrile electrolytes. The black arrow indicates the scan direction. Each CV is up to the stable double-layer voltage of each electrode–electrolyte system, demonstrating that the electrochemical stability is dependent on the identity of the cation and that the different $Cu_3(HHTP)_2$–electrolyte systems exhibit different stable double-layer voltage windows. This behaviour may reflect variations in the degree of electronic charging of the $Cu_3(HHTP)_2$ framework enabled by each electrolyte. Stronger interactions between smaller cations (e.g., Li^+) and the hydroxy-functionalised pore surface can increase local charge accumulation within the framework, potentially shifting the onset of irreversible framework degradation processes to lower cell voltages. For consistency, all capacities were measured at the maximum stable voltage of the least stable system, $Cu_3(HHTP)_2$ with Li^+, at 0.6 V. Further work is required to better understand the origin and mechanisms of degradation in these systems, including why higher charge density cations appear to accelerate degradation.

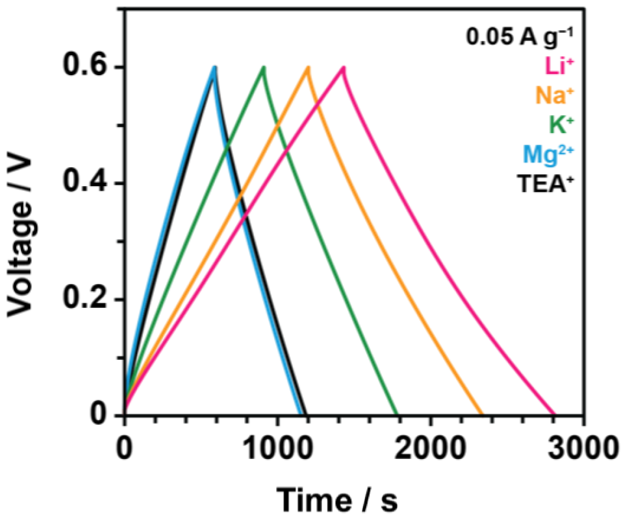

Figure S6: Galvanostatic charge-discharge (GCD) profiles at a current density of 0.05 A g $^{-1}$ from two-electrode symmetric supercapacitors assembled with $Cu_3(HHTP)_2$ electrodes (Sample 1) and 1 M solutions of LiTFSI, NaTFSI, KTFSI, TEATFSI, and $Mg(TFSI)_2$ in acetonitrile electrolytes. For consistency, all capacities were measured at the maximum stable voltage of the least stable system, $Cu_3(HHTP)_2$ with Li^+, at 0.6 V. Reported capacities were calculated from GCD discharge profiles.

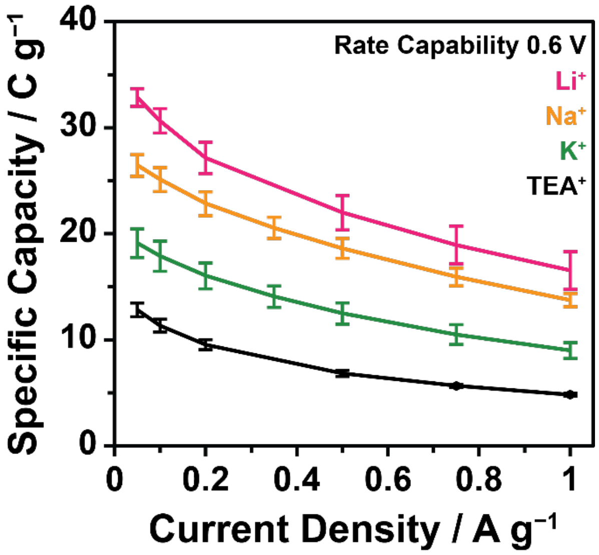

Figure S7: Capacity *vs*. current density plots from two-electrode symmetric supercapacitor cells assembled with $Cu_3(HHTP)_2$ electrodes, and 1 M solutions of LiTFSI, NaTFSI, KTFSI, and TEATFSI in acetonitrile electrolytes when charging to a cell voltage of 0.6 V. All specific capacity values were calculated from GCD experiments and the error bars are from two measurements on two independent cells with each electrolyte, each with a different sample of $Cu_3(HHTP)_2$ (Sample 1 and Sample 2).

Cation	Specific Capacity (0.6 V; 0.05 A g^{-1}) / C g^{-1}
Li^+	32.9 ± 1.7
Na^+	26.5 ± 2.1
K^+	19.1 ± 2.7
TEA^+	12.8 ± 1.3

Table S1: Specific capacity values for $Cu_3(HHTP)_2$ with a series of 1 M TFSI-based acetonitrile electrolytes with different cations, calculated from GCD experiments performed on symmetric two-electrode supercapacitor cells at a current density of 0.05 A g^{-1} when charging to a cell voltage of 0.6 V. This data is presented in Figure 2c of the Main Text.

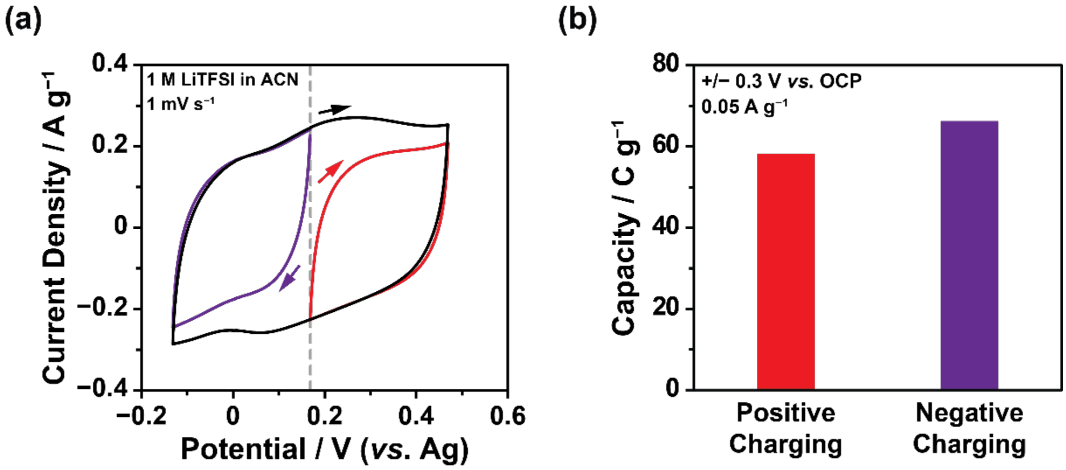

Figure S8: (a) CV data obtained at a scan rate of 1 mV s^{-1} from a three-electrode cell assembled with a $Cu_3(HHTP)_2$ working electrode, YP80F counter electrode (oversized by a factor of approximately 3), and Ag pseudo-reference electrode, with a 1 M solution of LiTFSI in acetonitrile electrolyte. The open circuit potential (OCP) is indicated by the grey dashed line. Data was acquired by scanning to +0.3 V *vs.* OCP (red), −0.3 V *vs.* OCP (purple), and across the full potential window (black). The scan direction is indicated by an arrow in each case. (b) Specific capacity values calculated from GCD profiles at a current density of 0.05 A g^{-1} when charging to +0.3 V *vs.* OCP (red) and −0.3 V *vs.* OCP (purple). The OCP was used as an internal reference as a clear potential of zero charge (PZC) could not be identified within the stable potential window of $Cu_3(HHTP)_2$ (measurements performed with 1 M $TEABF_4$ in acetonitrile; see SI Fig. S9).

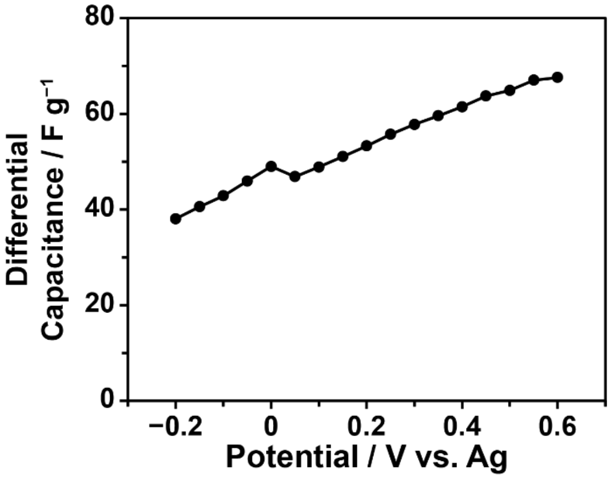

Figure S9: Differential capacitance vs. potential plot measured for $Cu_3(HHTP)_2$ in 1 M $TEABF_4$ in acetonitrile. As no minimum is observed across this potential range, no potential of zero charge (PZC) could be identified for this system.

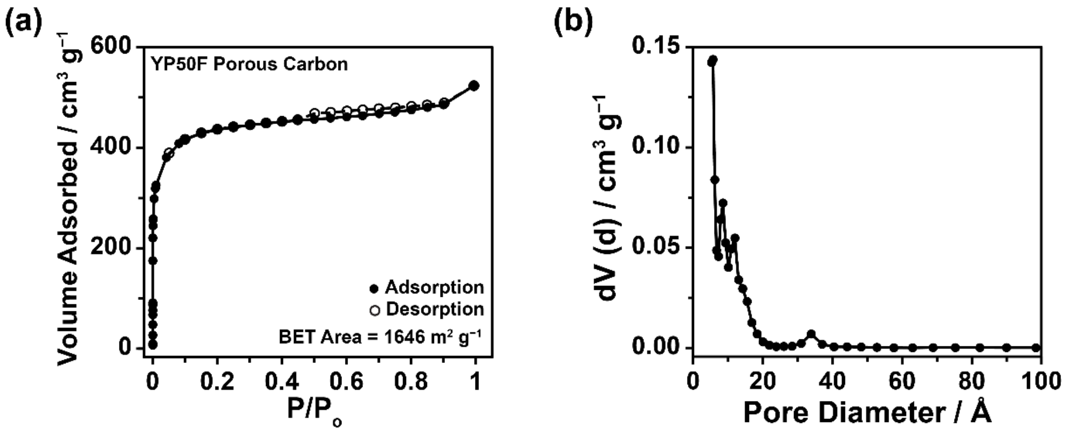

Figure S10: Characterisation data from a common porous carbon material, YP50F. (a) 77 K N_2 sorption isotherm. Adsorption data are shown with filled circles and a solid line, while desorption data are shown with unfilled circles and a dashed line. From this, a BET surface area of 1646 m^2 g^{-1} was determined. (b) Pore size distribution of YP50F, calculated from the N_2 sorption isotherm using a N_2 at 77 K on carbon (slit pores) quenched solid density functional theory (QSDFT) model. This shows that porous carbons typically have greater total porosities than layered MOFs, and more disordered pore structures.

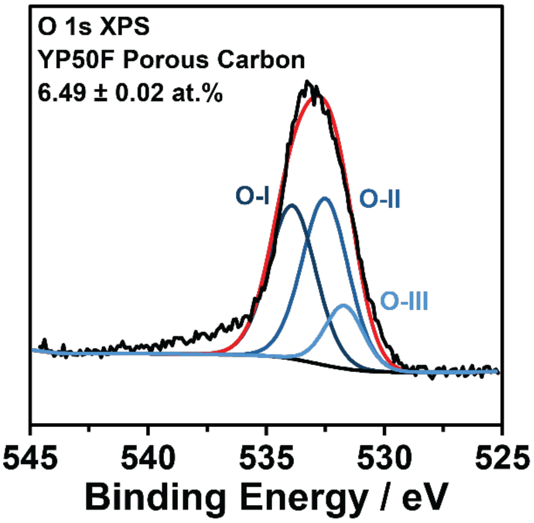

Figure S11: (a) Fitted O 1s XPS data obtained from YP50F porous carbon. From this, three main oxygen environments are found in this material: O-I represents C=O carbonyl groups, O-II represents C–OH hydroxyl or C–O–C ether groups, and O-III represents O=C–OH carboxyl groups. This shows that YP50F has a broad distribution of oxygen-based functional groups present. Adapted from Xu *et al* [1]

YP50F Carbon

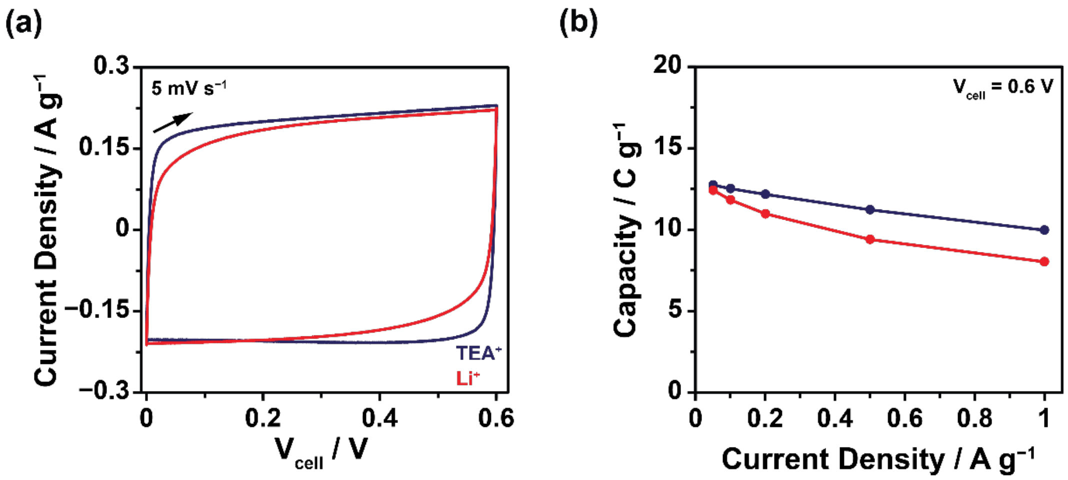

Figure S12: (a) CVs obtained at a scan rate of 5 mV s^{-1} up to 0.6 V for symmetric two-electrode supercapacitors assembled with YP50F film electrodes and 1 M solutions of TEATFSI (dark blue) and LiTFSI (red) in acetonitrile electrolytes. The black arrow indicates the scan direction. (b) Capacity *vs.* current density plots with charging up to 0.6 V for YP50F with each electrolyte. All capacity values were calculated from GCD experiments.

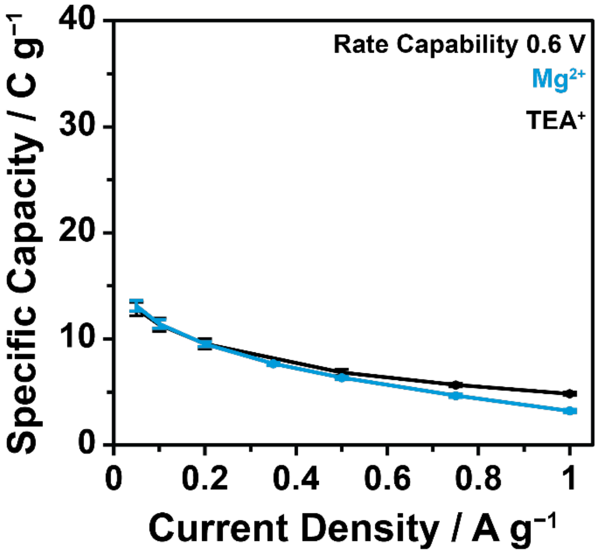

Figure S13: Capacity *vs*. current density plots from two-electrode symmetric supercapacitor cells assembled with $Cu_3(HHTP)_2$ electrodes, and 1 M solutions of TEATFSI and $Mg(TFSI)_2$ in acetonitrile electrolytes when charging to a cell voltage of 0.6 V. All specific capacity values were calculated from GCD experiments, and the error bars are from two measurements with each electrolyte with two different samples of $Cu_3(HHTP)_2$ (Sample 1 and Sample 2). This shows the similarity in performance between the two electrolytes.

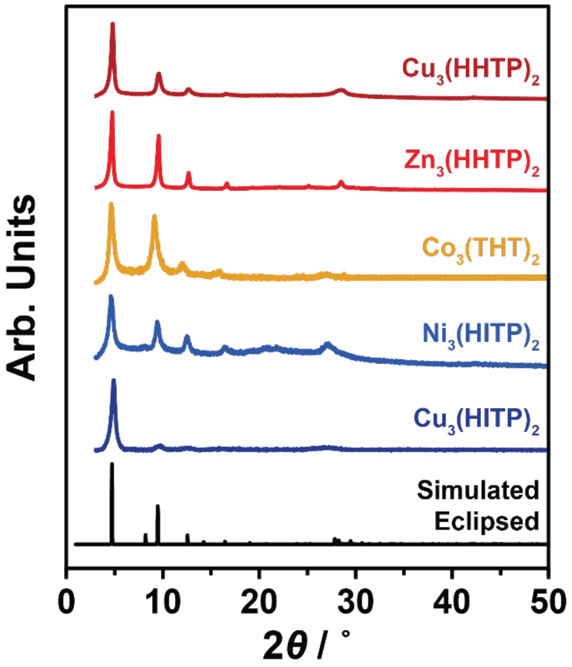

Figure S14: Experimental powder XRD patterns from each of the layered MOFs synthesised in this work, compared to the simulated XRD pattern for a layered triphenylene MOF with eclipsed layer stacking (black).

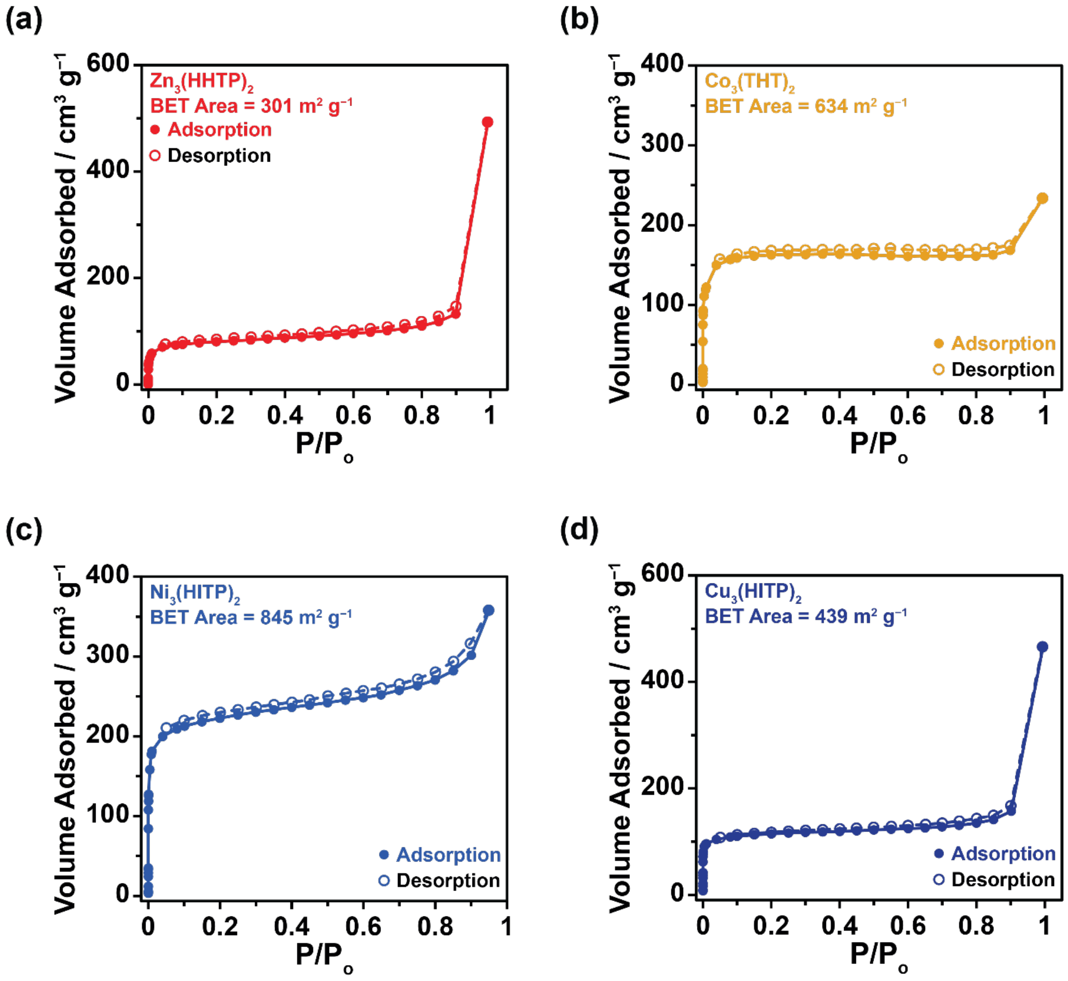

Figure S15: 77 K N_2 sorption isotherm of (a) $Zn_3(HHTP)_2$; (b) $Co_3(THT)_2$; (c) $Ni_3(HITP)_2$; (d) $Cu_3(HITP)_2$ synthesised in this work. Adsorption data are shown with filled circles, while desorption data are shown with unfilled circles. The calculated BET surface areas for each layered MOF are shown on each panel. The 77 K N_2 sorption isotherm of $Cu_3(HHTP)_2$ is shown in Figure S2.

MOF	BET Surface Area / $m^2\ g^{-1}$
$Cu_3(HHTP)_2$	875
$Zn_3(HHTP)_2$	301
$Co_3(THT)_2$	634
$Ni_3(HITP)_2$	845
$Cu_3(HITP)_2$	439

Table S2: BET surface areas of the layered MOFs synthesised in this work, calculated from 77 K N_2 sorption isotherms. This illustrates the difference in quality between the different MOFs. However, N_2 interactions via its quadrupole moment, along with the limitations of BET analysis in microporous materials, may affect the accuracy of absolute surface area values.[2,3]

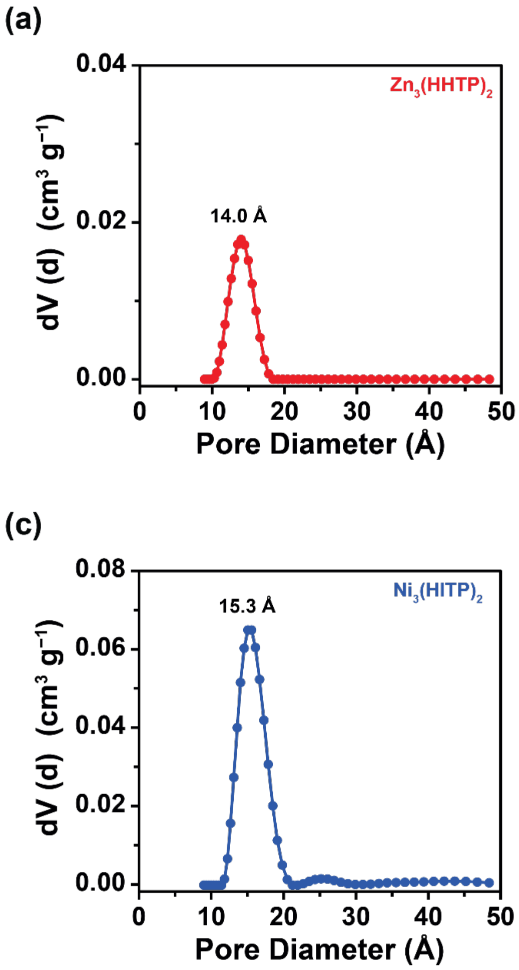

Figure S16: Pore size distributions for (a) $Zn_3(HHTP)_2$; (b) $Co_3(THT)_2$; (c) $Ni_3(HITP)_2$; (d) $Cu_3(HITP)_2$ synthesised in this work, calculated using a N_2 at 77 K on carbon (cylindrical pores) quenched solid density functional theory (QSDFT) model from the corresponding N_2 isotherms. While the results broadly align with crystallographically expected pore size of 15.0 Å, the application of carbon-based kernels to calculate the pore size distribution of MOFs can introduce deviations due to differences in pore geometry and surface chemistry. The pore size distribution of $Cu_3(HHTP)_2$ is shown in Figure S3.

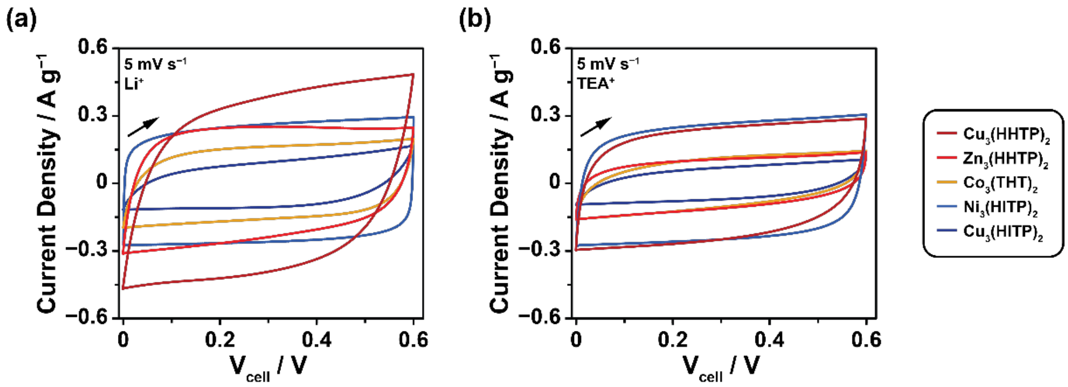

Figure S17: CVs obtained at a scan rate of 5 mV s^{-1} up to 0.6 V from two-electrode symmetric supercapacitors assembled with $Cu_3(HHTP)_2$ (dark red), $Zn_3(HHTP)_2$ (red), $Co_3(THT)_2$ (yellow), $Ni_3(HITP)_2$ (blue), and $Cu_3(HITP)_2$ (dark blue) electrodes and (a) 1 M LiTFSI in acetonitrile; (b) 1 M TEATFSI in acetonitrile electrolytes. The black arrows indicate the scan directions.

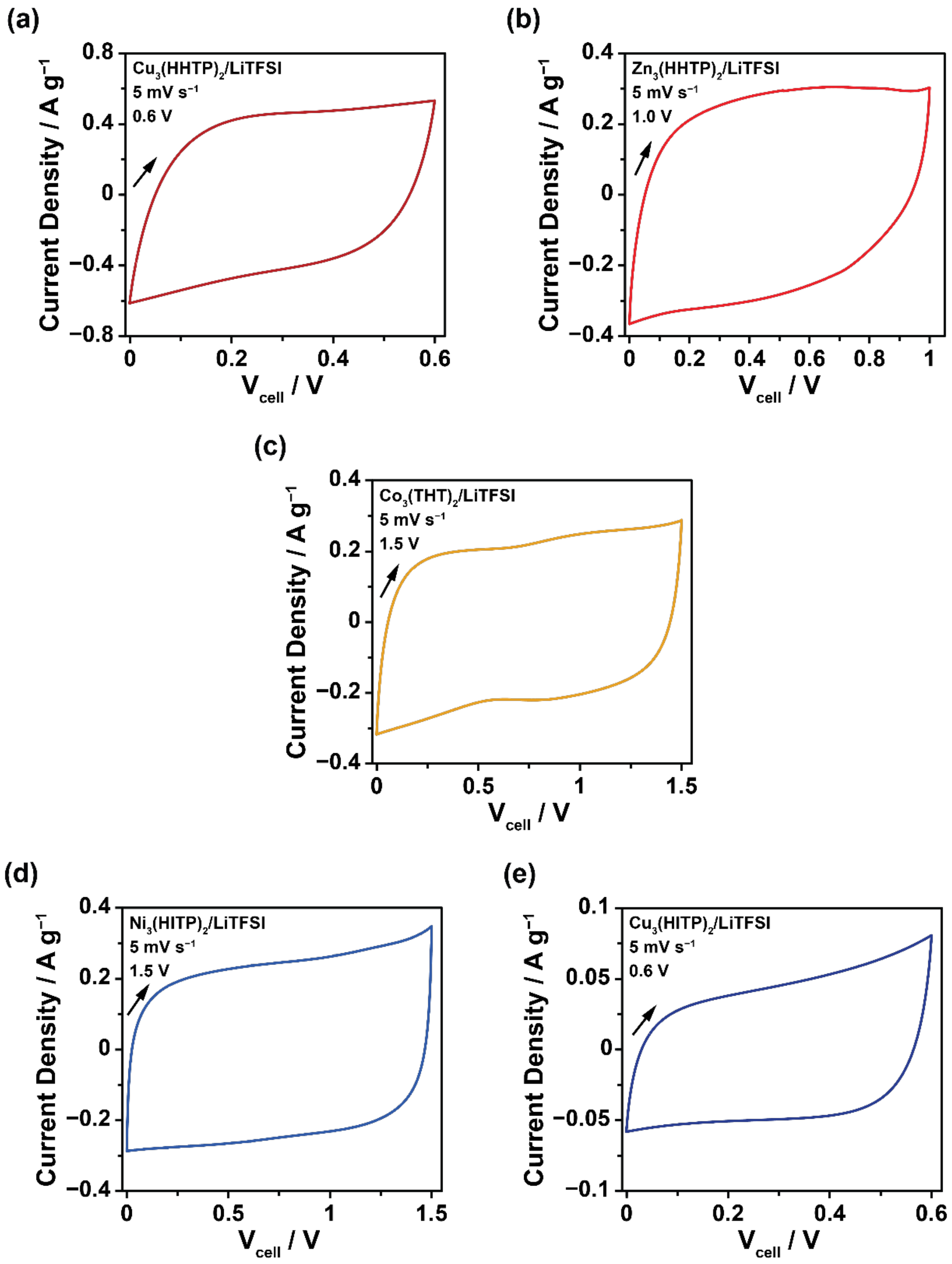

Figure S18: CVs obtained at a scan rate of 5 mV s^{-1} from two-electrode symmetric supercapacitors assembled with 1 M LiTFSI in acetonitrile electrolyte and (a) $Cu_3(HHTP)_2$; (b) $Zn_3(HHTP)_2$; (c) $Co_3(THT)_2$; (d) $Ni_3(HITP)_2$; and (e) $Cu_3(HITP)_2$ electrodes. Each CV is up to the stable double-layer voltage of each electrode–electrolyte system, demonstrating that the electrochemical stability is dependent on the identity of the metal–ligand functionality. The black arrows indicate the scan directions.

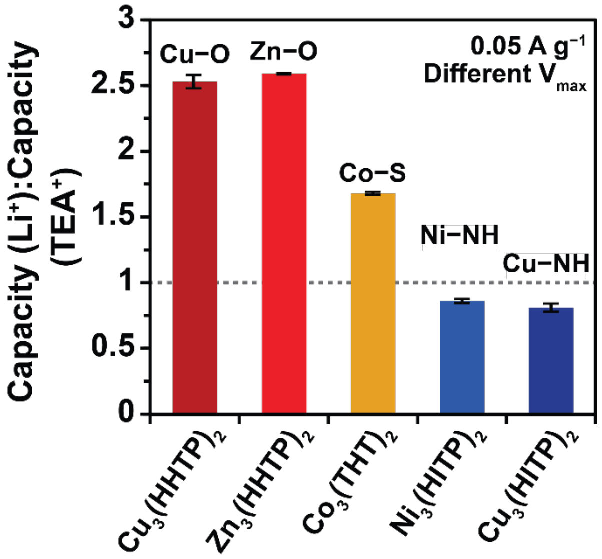

Figure S19: Capacity ratios (LiTFSI/TEATFSI) in 1 M acetonitrile electrolytes for a series of layered MOFs with different combinations of metal node and ligating group, calculated from GCD experiments on symmetric two-electrode supercapacitor cells at a current density of 0.05 A g^{-1}. Voltage limits were selected based on the electrochemical stability of each system. Ratios for $Cu_3(HHTP)_2$ and $Cu_3(HITP)_2$ were calculated at 0.6 V (as in Figure 3b; Main Text), while those for $Zn_3(HHTP)_2$, $Co_3(THT)_2$, and $Ni_3(HITP)_2$ were calculated at 1.0 V. $Zn_3(HHTP)_2$ showed ~2.5× increase in capacity with Li^+, $Co_3(THT)_2$ showed a ~1.7× increase, and no enhancement was observed for the $Ni_3(HITP)_2$. This shows that consistent capacity ratio trends are observed across different voltage windows. Error bars represent measurements from at least two independent MOF batches per system.

MOF	Capacity (TEA^+) / C g^{-1}	Capacity (Li^+) / C g^{-1}
$Cu_3(HHTP)_2$ (0.6 V)	12.5 ± 1.2	31.5 ± 2.3
$Zn_3(HHTP)_2$ (1 V)	13.5 ± 0.4	34.9 ± 1.3
$Co_3(THT)_2$ (1 V)	12.0 ± 0.5	20.1 ± 0.3
$Ni_3(HITP)_2$ (1 V)	31.7 ± 1.5	27.1 ± 0.4
$Cu_3(HITP)_2$ (0.6 V)	10.5 ± 1.2	8.6 ± 1.8

Table S3: Specific capacity values for the series of layered MOFs synthesised in this work with both 1 M TEATFSI and 1 M LiTFSI in acetonitrile electrolytes, calculated from GCD experiments performed on symmetric two-electrode supercapacitor cells at a current density of 0.05 A g^{-1}. The voltage limit varied depending on the stability of the systems and is given in the Table. This data was used to calculate the capacity ratios presented in SI Figure S18.

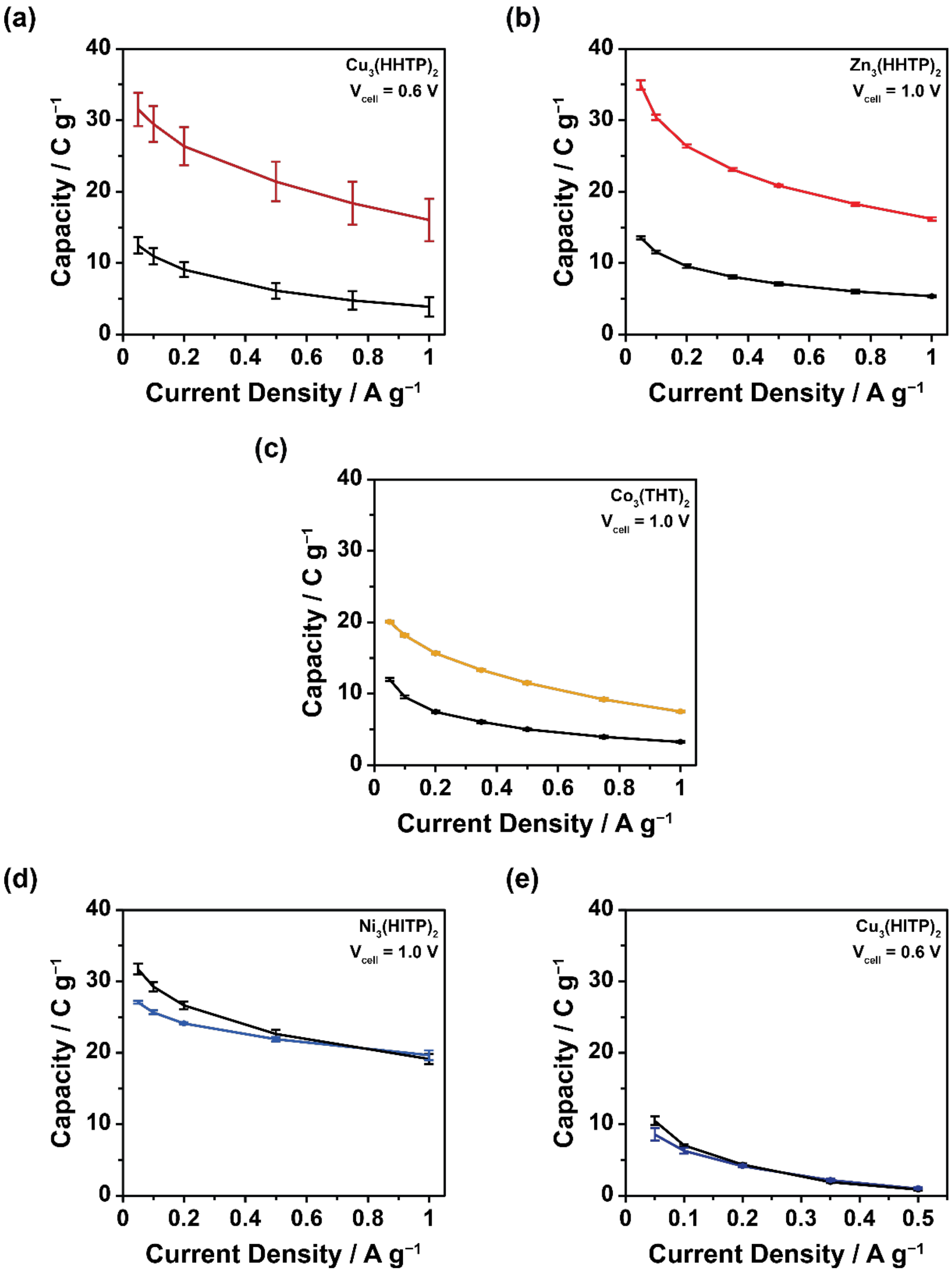

Figure S20: Capacity *vs*. current density plots from (a) $Cu_3(HHTP)_2$; (b) $Zn_3(HHTP)_2$; (c) $Co_3(THT)_2$; (d) $Ni_3(HITP)_2$; and (e) $Cu_3(HITP)_2$ with both 1 M LiTFSI in acetonitrile (colour datasets) and 1 M TEATFSI in acetonitrile (black datasets) electrolytes. All specific capacity values were calculated from GCD experiments, and error bars represent measurements from at least two independent MOF batches per system. The voltage limit used varied depending on the stability of the systems and is given on the top right of the panel.

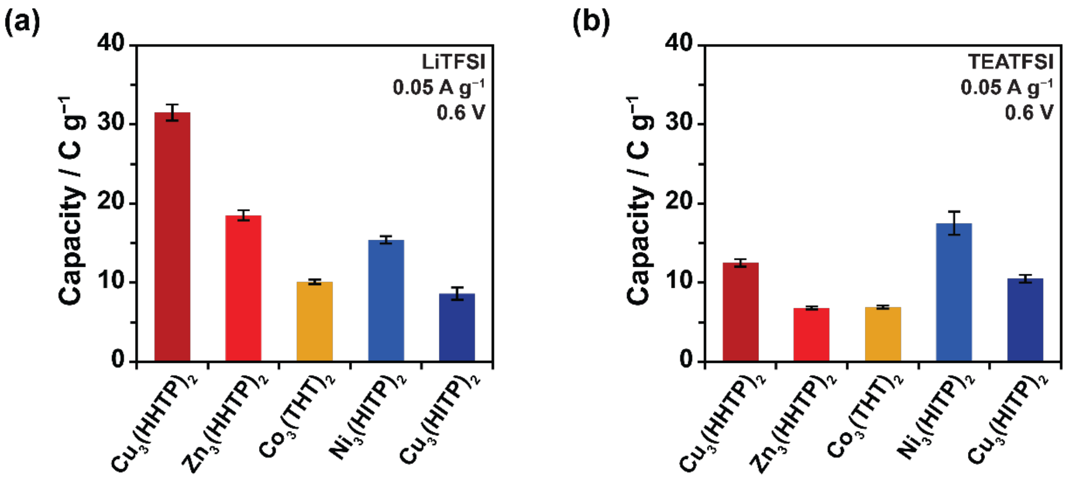

Figure S21: Specific capacity values for the series of layered MOFs synthesised in this work with (a) 1 M LiTFSI in acetonitrile electrolyte; and (b) 1 M TEATFSI in acetonitrile electrolyte. All values were calculated from GCD experiments performed on symmetric two-electrode supercapacitor cells at a current density of 0.05 A g^{-1} when charging to a cell voltage of 0.6 V. The error bars are from two measurements on each system with different MOF batches. This illustrates the significant variation in the absolute electrochemical capacities of the different layered MOFs.

MOF	Capacity (TEA^+) / C g^{-1}	Capacity (Li^+) / C g^{-1}
$Cu_3(HHTP)_2$	12.5 ± 1.2	31.5 ± 2.3
$Zn_3(HHTP)_2$	6.8 ± 0.6	18.5 ± 1.5
$Co_3(THT)_2$	6.9 ± 0.6	10.1 ± 0.8
$Ni_3(HITP)_2$	17.5 ± 3.2	15.4 ± 1.2
$Cu_3(HITP)_2$	10.5 ± 1.2	8.6 ± 1.8

Table S4: Specific capacity values for the series of layered MOFs synthesised in this work with both 1 M TEATFSI and 1 M LiTFSI in acetonitrile electrolytes, calculated from GCD experiments performed on symmetric two-electrode supercapacitor cells at a current density of 0.05 A g^{-1} when charging to 0.6 V. This data was used to calculate the capacity ratios presented in Figure 3b of the Main Text.

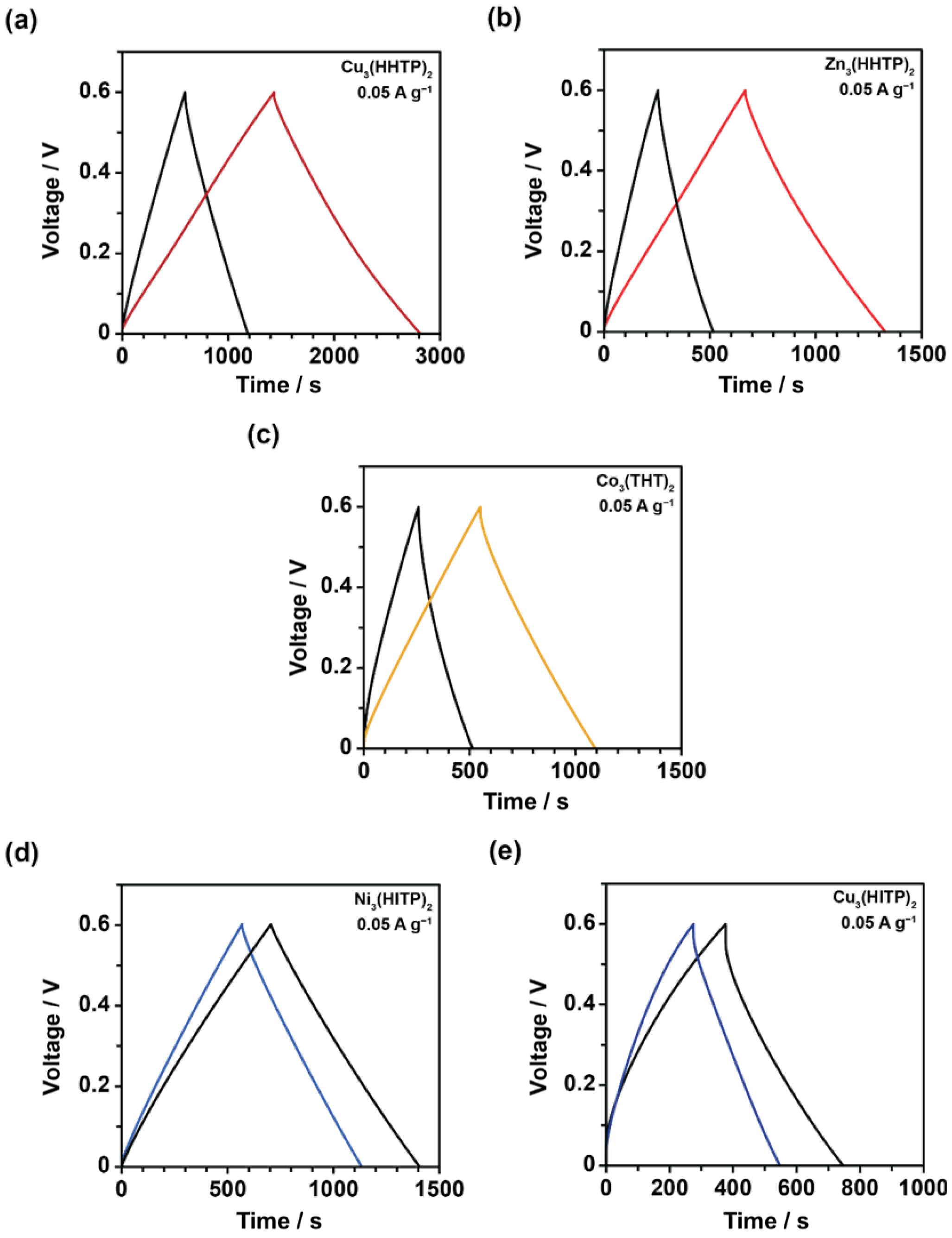

Figure S22: Galvanostatic charge-discharge (GCD) profiles at a current density of 0.05 A g $^{-1}$ from two-electrode symmetric supercapacitors assembled with (a) $Cu_3(HHTP)_2$; (b) $Zn_3(HHTP)_2$; (c) $Co_3(THT)_2$; (d) $Ni_3(HITP)_2$; and (e) $Cu_3(HITP)_2$ electrodes with both 1 M LiTFSI in acetonitrile (colour datasets) and 1 M TEATFSI in acetonitrile (black datasets) electrolytes. For consistency, all GCDs are reported when charging to 0.6 V. Reported capacities were calculated from GCD discharge profiles.

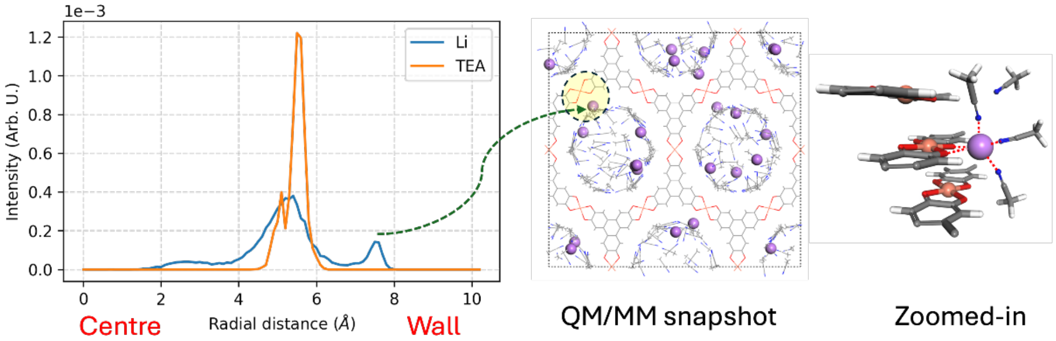

Figure S23: QM/MM simulation results showing cation localisation in $Cu_3(HHTP)_2$ with Li^+ and TEA^+ electrolytes. (a) Radial distribution profiles showing the position of Li^+ (blue) and TEA^+ (orange) cations relative to the pore wall in $Cu_3(HHTP)_2$. (b) Snapshot from QM/MM simulation showing Li^+ cation positions (purple) within the MOF pores. (c) Representative coordination geometry of a Li^+ cation near the pore wall, interacting with deprotonated oxygen atoms of the HHTP ligands.

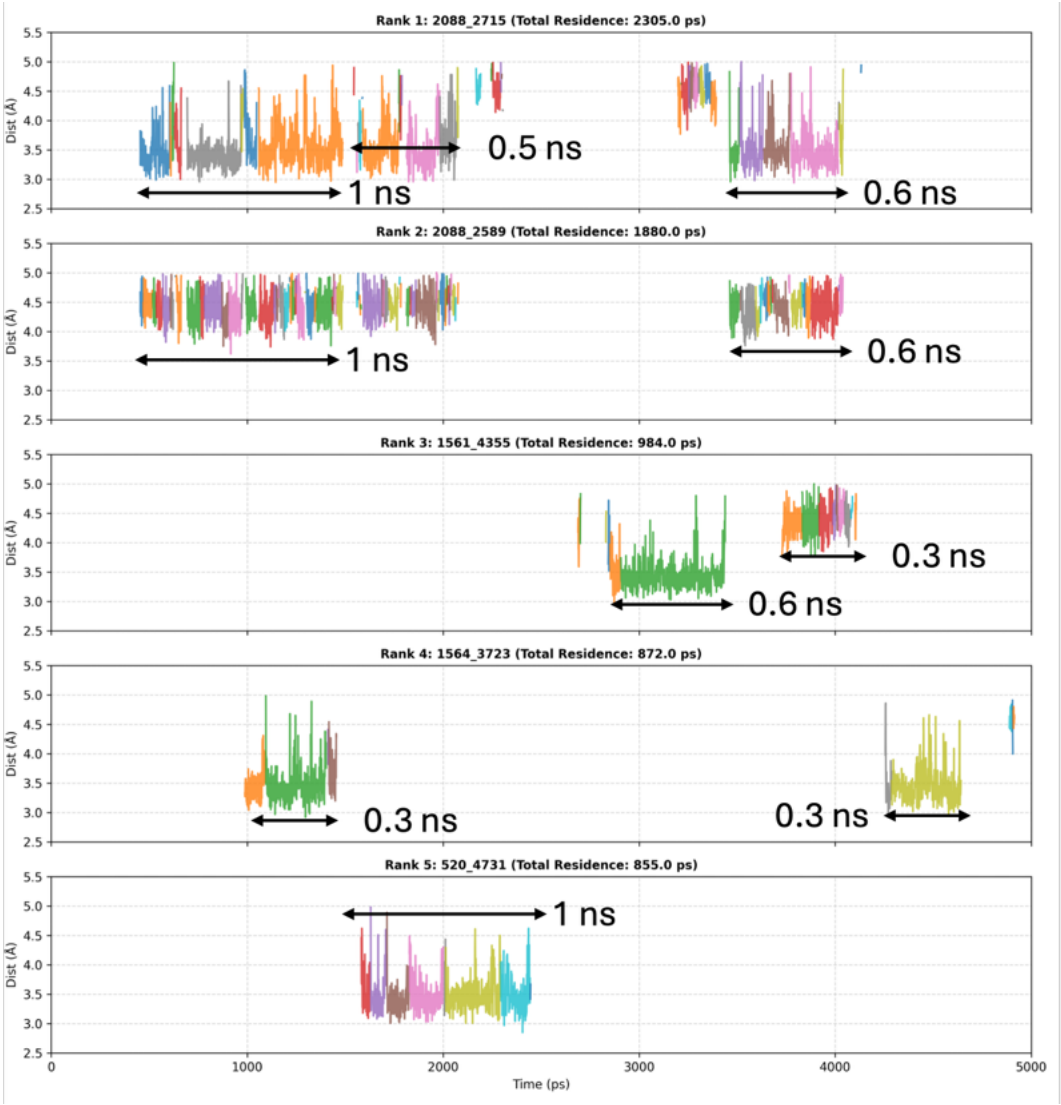

Figure S24: Interfacial distance analysis between Li^+ and Cu–O_4 coordination sites. This distance is defined as an average distance of two bonds between the Li^+ and the two accessible oxygen atoms of the Cu-O_4 complex. The top five pairs, ranked by cumulative residence time, are displayed. Distinct colors indicate transient de-coordination events. A cutoff distance of 5 Å was applied to define the coordination state.

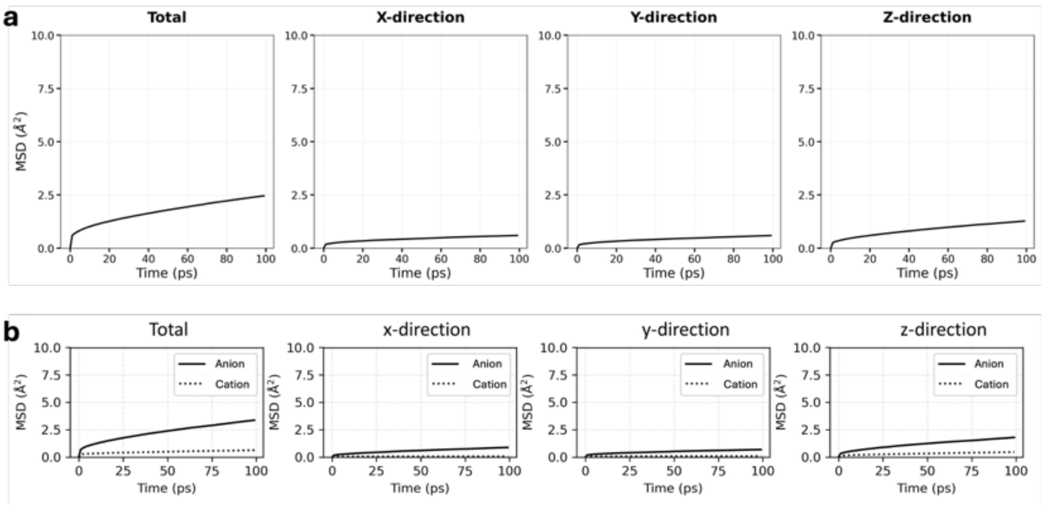

Figure S25: Mean squared displacement (MSD) of electrolyte ions in the pores of $Cu_3(HHTP)_2$. (a) Li^+ and (b) TEA^+ ions are compared. Data for TEA^+ has been taken from a previous study[4]

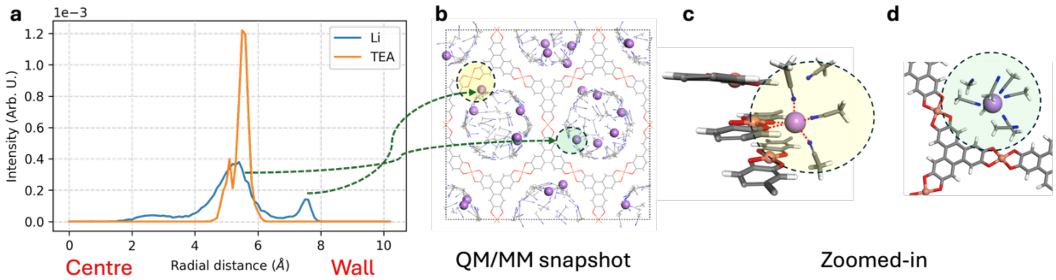

Figure S26: QM/MM simulation results showing cation localisation in negatively charged $Cu_3(HHTP)_2$ with Li^+ and TEA^+ electrolytes. (a) Radial distribution profiles showing the position of Li^+ (blue) and TEA^+ (orange) cations relative to the pore wall in $Cu_3(HHTP)_2$. (b) Snapshot from QM/MM simulation showing Li^+ cation positions (purple) within the MOF pores. (c), (d) Representative coordination geometry of a Li^+ cation near the pore wall, interacting with deprotonated oxygen atoms of the HHTP ligands (c) and a non-interacting state (d).

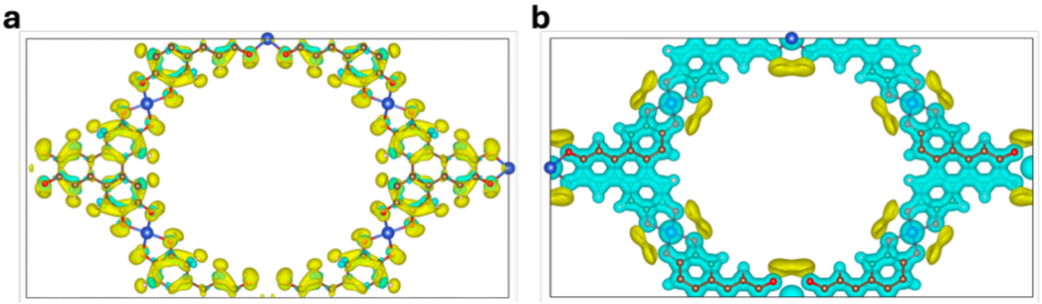

Figure S27: Isosurface plots from QM/MM simulations. (a) Charge density difference isosurface for the charged interface with Li^+. The electrolyte region is omitted for clarity. Isosurface level: $3{\times}10^{-4}$ e $bohr^{-3}$. (b) Electrostatic potential isosurface of the charged interface. Isosurface level: 0.38 Rydberg.

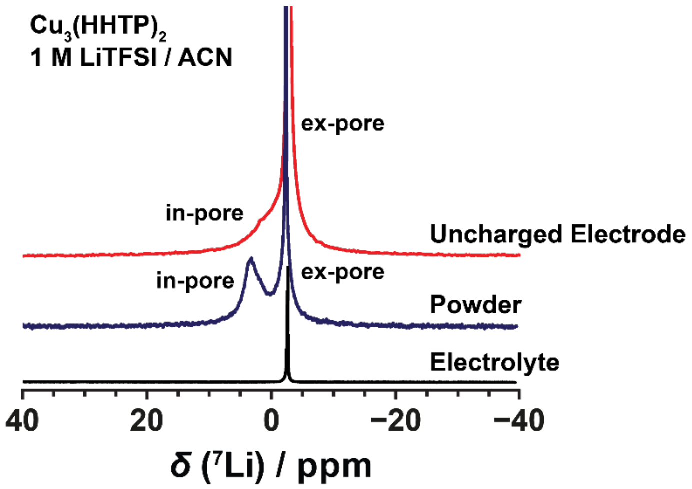

Figure S28: ^{7}Li solid-state NMR (9.4 T; 25 kHz magic-angle spinning) experiments of both $Cu_3(HHTP)_2$ powder soaked with 1 M LiTFSI in acetonitrile electrolyte (dark blue) and ex situ $Cu_3(HHTP)_2$ electrodes extracted from a symmetric two-electrode supercapacitor assembled with 1 M LiTFSI in acetonitrile held at 0 V for 1.5 h (red). Spectra are normalised to the mass of MOF in each sample. The black spectrum (bottom) corresponds to the neat electrolyte.

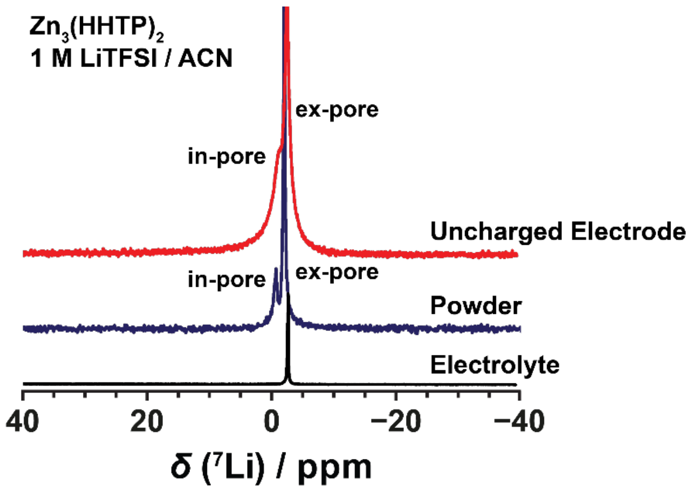

Figure S29: ^{7}Li solid-state NMR (9.4 T; 25 kHz magic-angle spinning) experiments of both $Zn_3(HHTP)_2$ powder soaked with 1 M LiTFSI in acetonitrile electrolyte (dark blue) and ex situ $Zn_3(HHTP)_2$ electrodes extracted from a symmetric two-electrode supercapacitor assembled with 1 M LiTFSI in acetonitrile held at 0 V for 1.5 h (red). Spectra are normalised to the mass of MOF in each sample. The black spectrum (bottom) corresponds to the neat electrolyte.

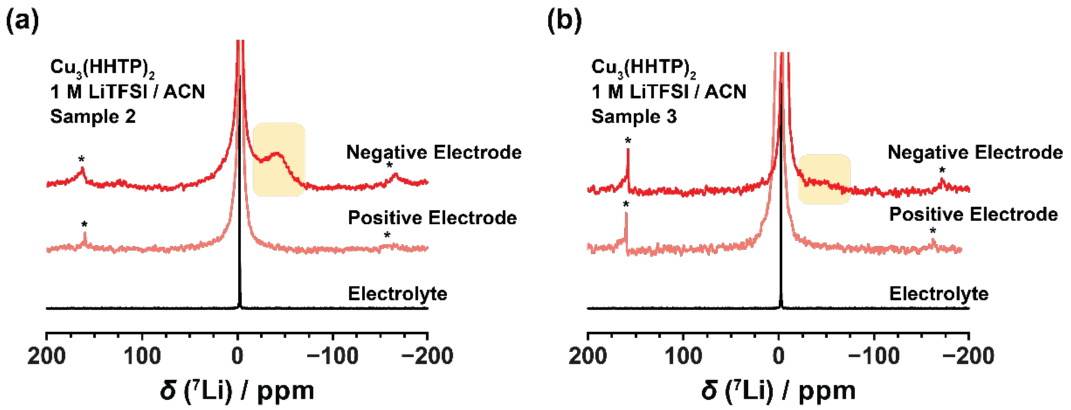

Figure S30: Repeat ^{7}Li solid-state ex situ NMR (9.4 T; 25 kHz magic-angle spinning) experiments of charged $Cu_3(HHTP)_2$ electrodes extracted from symmetric two-electrode supercapacitors assembled with 1 M LiTFSI in acetonitrile from two additional samples of $Cu_3(HHTP)_2$ (a) Sample 2; and (b) Sample 3. Spectra are normalised to the mass of MOF in each sample. The black spectrum (bottom) corresponds to the neat electrolyte. Asterisks denote spinning sidebands. This shows the variability in the intensity of the negatively shifted peak with MOF sample.

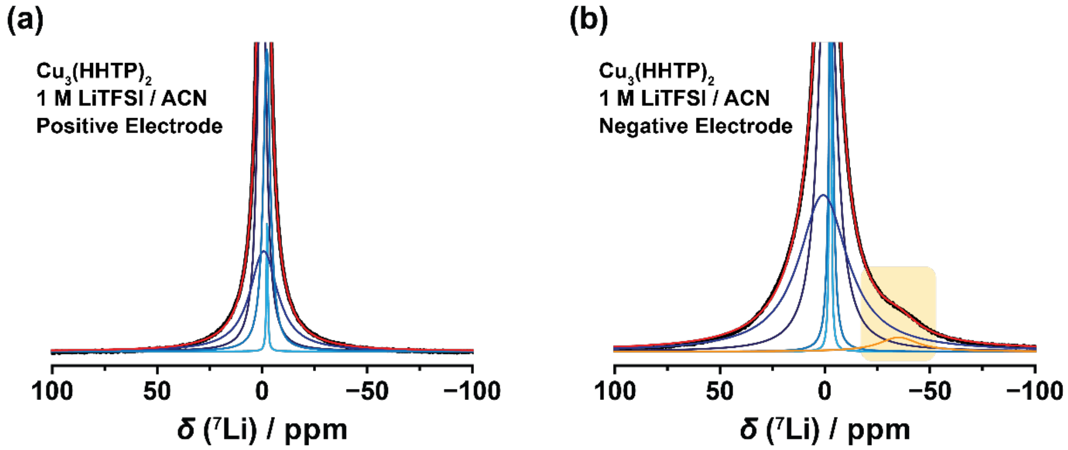

Figure S31: Examples of fittings for ^{7}Li solid-state ex situ NMR spectra (9.4 T; 25 kHz magic-angle spinning) of charged $Cu_3(HHTP)_2$ electrodes extracted from symmetric two-electrode supercapacitors assembled with 1 M LiTFSI in acetonitrile. Data are shown for both the (a) positive and (b) negative electrodes. The experimental spectrum is shown in black, the overall fit in red, and fitted peaks common to both electrodes in shades of blue. The negative electrode in (b) exhibits an additional feature at negative shifts (orange; highlighted by the yellow box). All spectra were recorded with a short recycle delay (0.1 s) to accentuate any paramagnetic shifts.

Peak	Chemical Shift / ppm	Peak Width / ppm	G/L	CSA / ppm	η
		**Positive Electrode**			
1	−2.2	0.7	0	129	0
2	−2.1	4.0	0	N/A	N/A
3	−0.7	16.0	0	129	0.25
4	0.2	4.0	0	−96	0
		**Negative Electrode**			
1	−2.5	0.4	0	−64	0
2	−2.8	2.3	0	80	0
3	0.9	28.0	0	−192	0
4	−1.0	8.6	0	112	0.75
5	−35.0	23.0	0	350	0

Table S5: Approximate fitting parameters for the two example ^{7}Li NMR fitted spectra shown in SI Figure S24, corresponding to the positive and negative $Cu_3(HHTP)_3$ electrodes. These results are intended to provide illustrative examples of component environments rather than exact quantification.

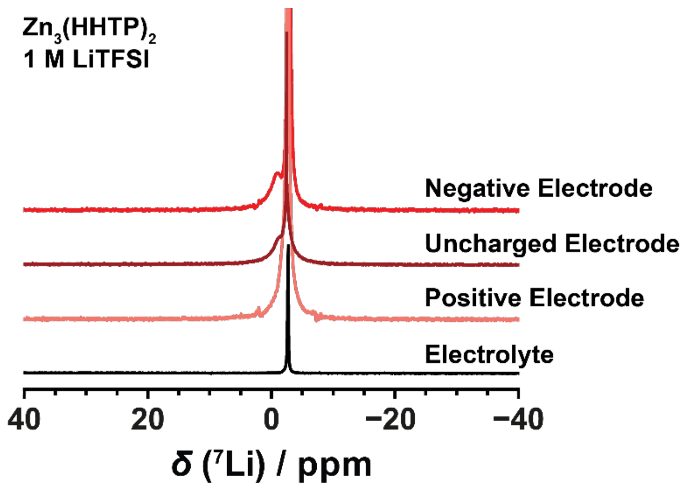

Figure S32: ^{7}Li solid-state ex situ NMR (9.4 T; 25 kHz magic-angle spinning) experiments of charged and uncharged $Zn_3(HHTP)_2$ electrodes extracted from symmetric two-electrode supercapacitors assembled with 1 M LiTFSI in acetonitrile. The positive and negative electrodes were extracted from a supercapacitor cell held at 0.5 V for 1 h (light red and red spectra). The uncharged electrode was extracted from a supercapacitor cell held at 0 V for 1.5 h (dark red spectrum). Spectra are normalised to the mass of MOF in each sample. The black spectrum (bottom) corresponds to the neat electrolyte.

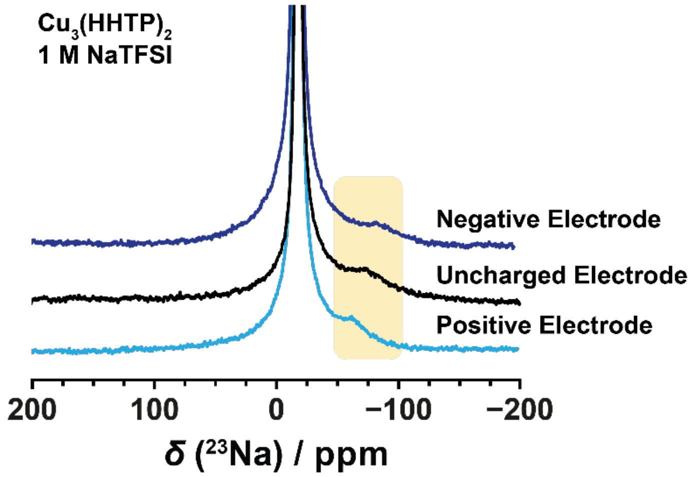

Figure S33: ^{23}Na solid-state ex situ NMR (9.4 T; 25 kHz magic-angle spinning) experiments of $Cu_3(HHTP)_2$ electrodes extracted from symmetric two-electrode supercapacitors assembled with 1 M NaTFSI in acetonitrile. Spectra are normalised to the mass of MOF in each sample. The positive and negative electrodes were extracted from a supercapacitor cell held at 0.5 V for 1 h (dark blue and light blue spectra). The uncharged electrode was extracted from a supercapacitor cell held at 0 V for 1.5 h (black spectrum).

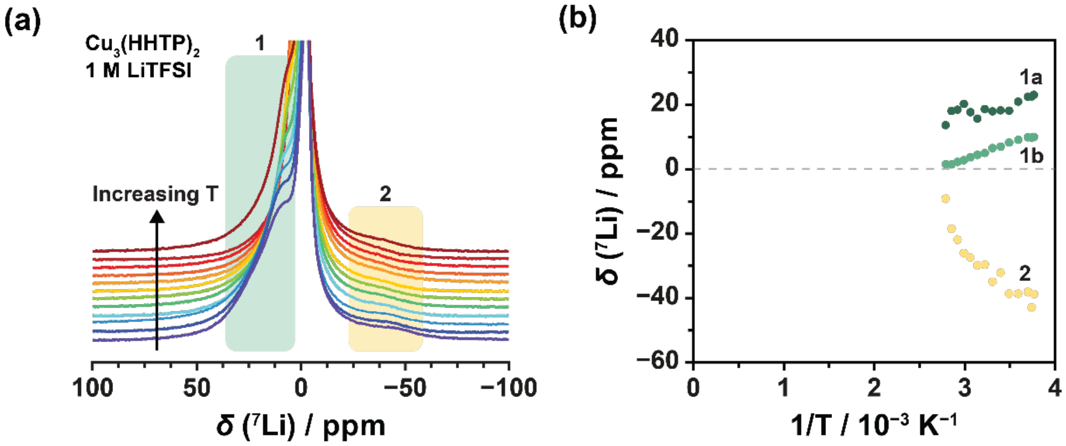

Figure S34: (a) ^{7}Li solid-state ex situ variable temperature NMR (9.4 T; 25 kHz magic-angle spinning) experiments of a negatively charged $Cu_3(HHTP)_2$ electrode extracted from a symmetric two-electrode supercapacitors assembled with 1 M LiTFSI in acetonitrile and held at 0.5 V for 1 h. Spectra are increasing in temperature from bottom (−7.3 °C; purple) to top (85.5 °C; red), as indicated by the black arrow. Spectra are recorded with a short recycle delay to accentuate the paramagnetic shift. The green box (1) highlights the positively shifted paramagnetic feature, while the yellow box (2) highlights the negatively shifted paramagnetic feature. (b) Plots of the chemical shift, $\delta(^7Li)$, for NMR environments 1 and 2 as a function of inverse temperature. Chemical shifts were extracted from spectral fitting, with environment 1 further resolved into two components (1a and 1b). All environments exhibited temperature-dependent shifts, with peak positions moving progressively toward the diamagnetic region at higher temperatures.

Cu−O−Li Angle / °	No. Solvent Molecules	Fermi Contact Shift, δ^{7Li}_{node} / ppm
91.6	2	−34
119.1	3	+9

Table S6: Hybrid DFT calculations of ^{7}Li Fermi contact NMR shifts of Li^{+} ions in $Cu_3(HHTP)_2$ structures with different binding geometries and numbers of acetonitrile molecules at 320 K.

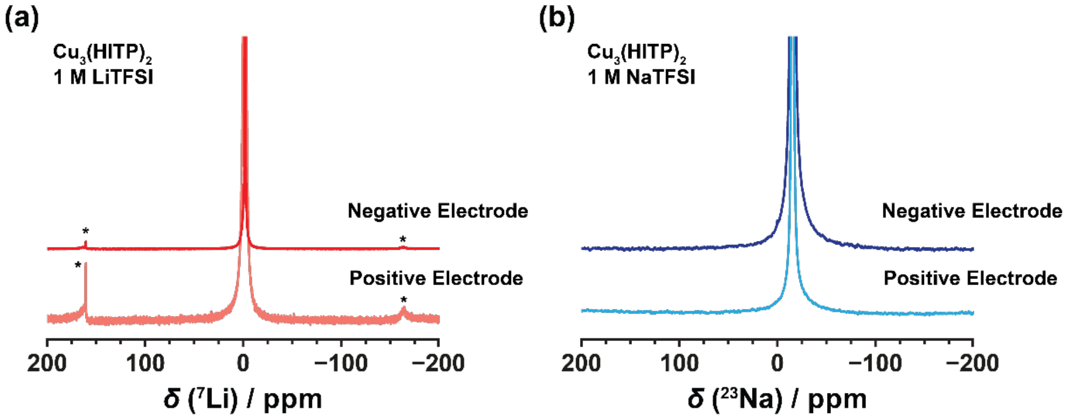

Figure S35: Solid-state ex situ NMR (9.4 T; 25 kHz magic-angle spinning) experiments of charged $Cu_3(HITP)_2$ electrodes extracted from symmetric two-electrode supercapacitors assembled with (a) 1 M LiTFSI (^7Li NMR); and (b) 1 M NaTFSI (^{23}Na NMR) in acetonitrile electrolytes. Spectra are normalised to the mass of MOF in each sample. All spectra are recorded with a short recycle delay to accentuate any paramagnetic shifts. Asterisks denote spinning sidebands. This confirms that no negatively shifted peaks are seen in the NMR spectra of $Cu_3(HITP)_2$ following charging.

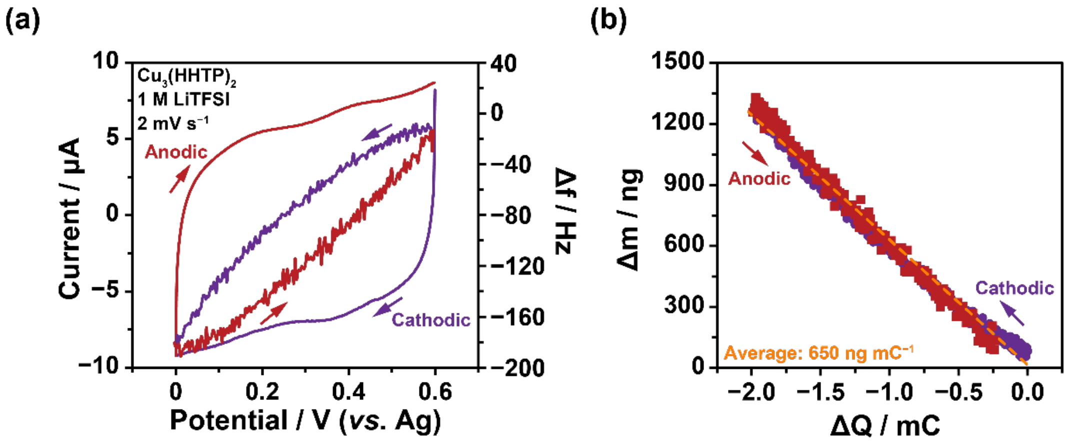

Figure S36: (a) CV and EQCM frequency response of $Cu_3(HHTP)_2$ with 1 M LiTFSI in acetonitrile electrolyte, obtained at a scan rate of 2 mV s^{-1} in the potential range from 0 to +0.6 V *vs*. Ag. The scan direction is indicated by an arrow in each case. (b) Plot of electrode mass change, (Δm), calculated from the frequency response shown in (a), against accumulated charge (ΔQ). ΔQ was calculated by integrating the current against time for the CV and setting the charge at the electrode potential of +0.6 V *vs*. Ag to zero. The dashed line (orange) shows the average mass change during the full CV experiment.

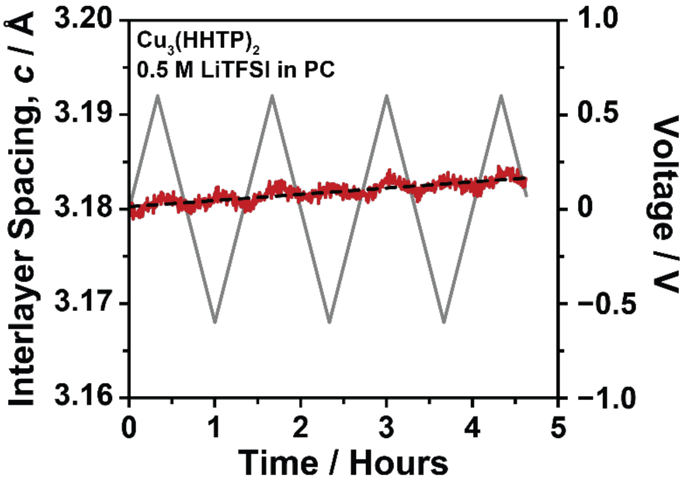

Figure S37: Variation in the interlayer spacing, *c*, (dark red line) as a function of applied cell voltage (grey line) obtained from operando wide angle X-ray scattering (WAXS) measurements on a custom-made symmetric two-electrode supercapacitor cell assembled with $Cu_3(HHTP)_2$ electrodes and 0.5 M LiTFSI in propylene carbonate electrolyte. The dashed line (black) shows the overall trend in the interlayer spacing during electrochemical cycling.

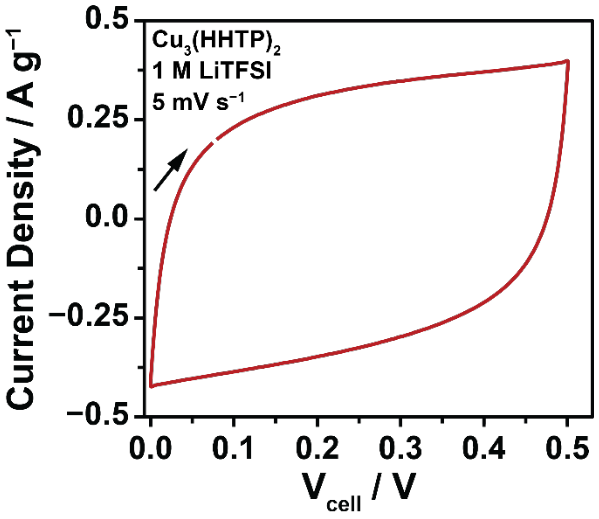

Figure S38: CV recorded at a scan rate of 5 mV s^{-1} up to 0.5 V from a custom-built AMPIX symmetric two-electrode supercapacitor assembled with $Cu_3(HHTP)_2$ film electrodes and 1 M LiTFSI in acetonitrile. The black arrow indicates the scan direction. The quasi-rectangular shape confirms the high capacitive performance of $Cu_3(HHTP)_2$ in the cell used for operando XRD measurements.

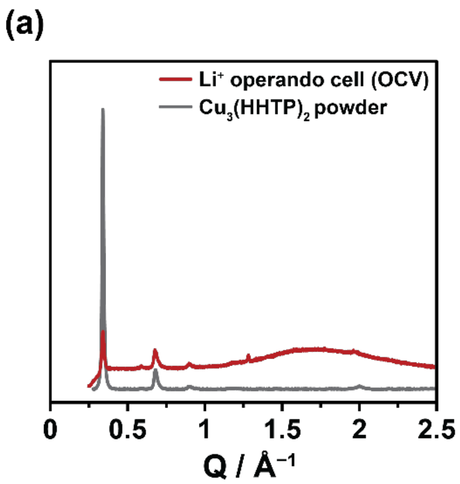

Figure S39: (a) Experimental synchrotron XRD patterns from $Cu_3(HHTP)_2$ powder (grey) and $Cu_3(HHTP)_2$ electrodes assembled in an operando symmetric two-electrode supercapacitor with 1 M LiTFSI in acetonitrile (dark red). (b) Zoomed-in view of the (001) reflection. The small rightward shift in the position of the (001) peak indicates an increase in the interlayer spacing of $Cu_3(HHTP)_2$ upon cell assembly with LiTFSI, consistent with wetting-induced electrode swelling.[5]

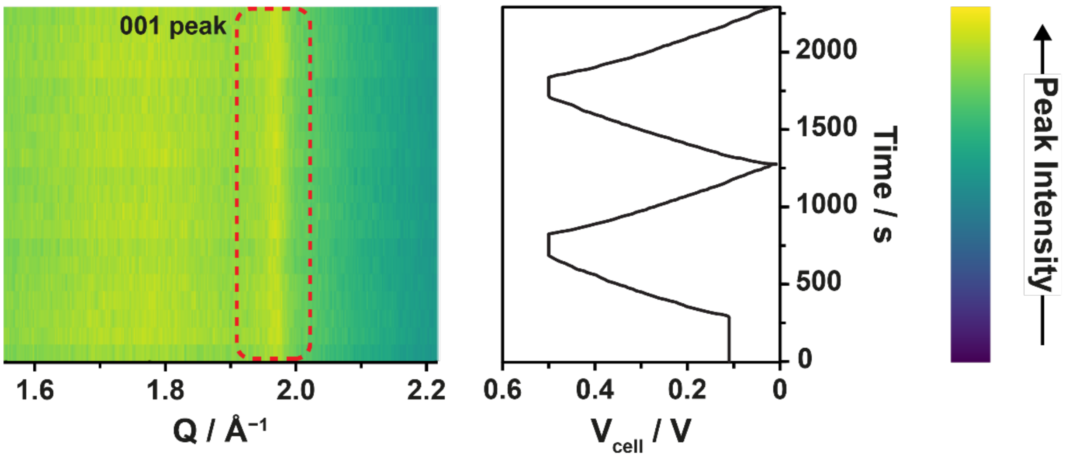

Figure S40: Operando XRD map of $Cu_3(HHTP)_2$ electrodes in a symmetric two-electrode supercapacitor assembled with 1 M LiTFSI in acetonitrile. The (001) reflection, corresponding to the interlayer spacing, is highlighted by the red dashed box. The applied charging protocol is shown alongside and is time-aligned with the XRD data. XRD data was acquired in transmission mode. Minimal variation in the interlayer spacing is observed during electrochemical cycling.

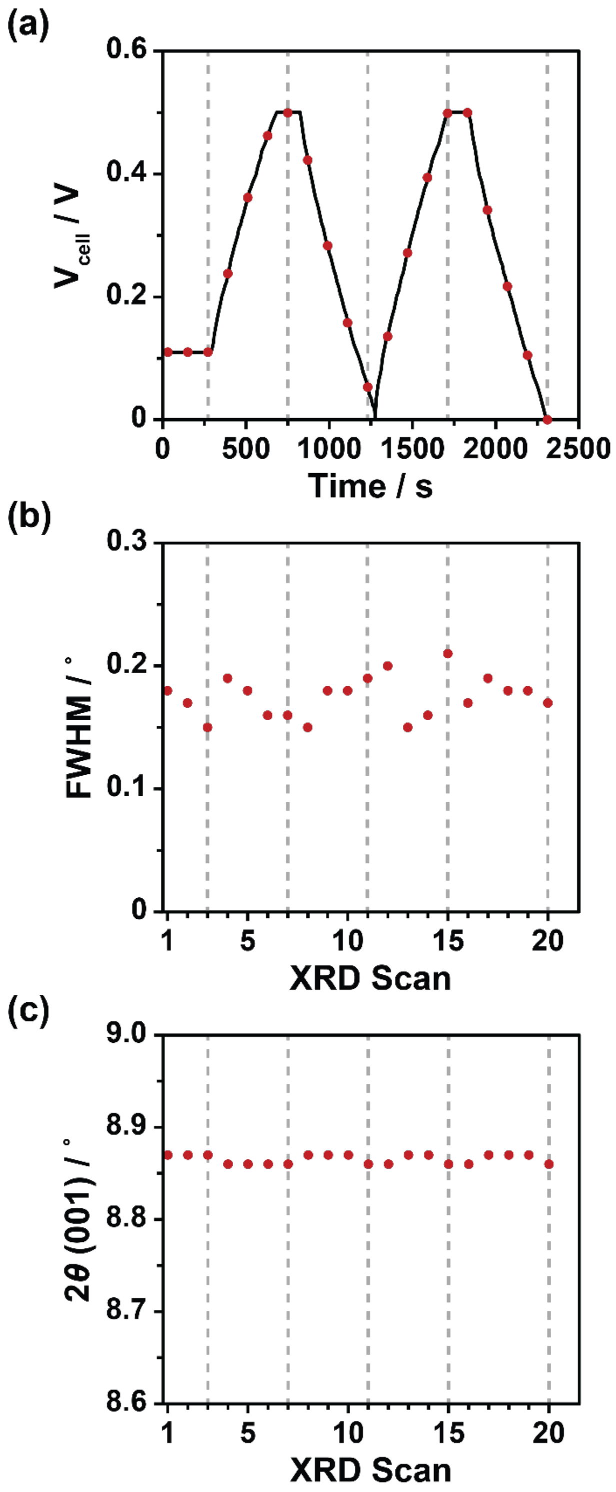

Figure S41: (a) Electrochemical voltage profile from operando XRD measurements of $Cu_3(HHTP)_2$ with 1 M LiTFSI in acetonitrile. Red markers indicate the times at which individual XRD scans were collected, with scans collected every 180 s. (b) Full width at half maximum (FWHM) of the (001) reflection as a function of scan number, showing minimal broadening and no correlation with applied cell voltage throughout the experiment. (c) Position of the (001) reflection (2θ) over the same series of scans, showing no significant shift in interlayer spacing during electrochemical cycling.

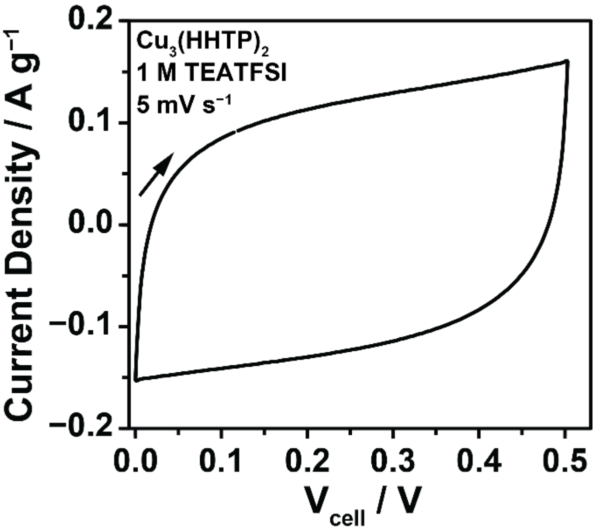

Figure S42: CV recorded at a scan rate of 5 mV s^{-1} up to 0.5 V from a custom-built AMPIX symmetric two-electrode supercapacitor assembled with $Cu_3(HHTP)_2$ film electrodes and 1 M TEATFSI in acetonitrile. The black arrow indicates the scan direction. The quasi-rectangular shape confirms the high capacitive performance of $Cu_3(HHTP)_2$ with this electrolyte in the cell used for operando XRD measurements.

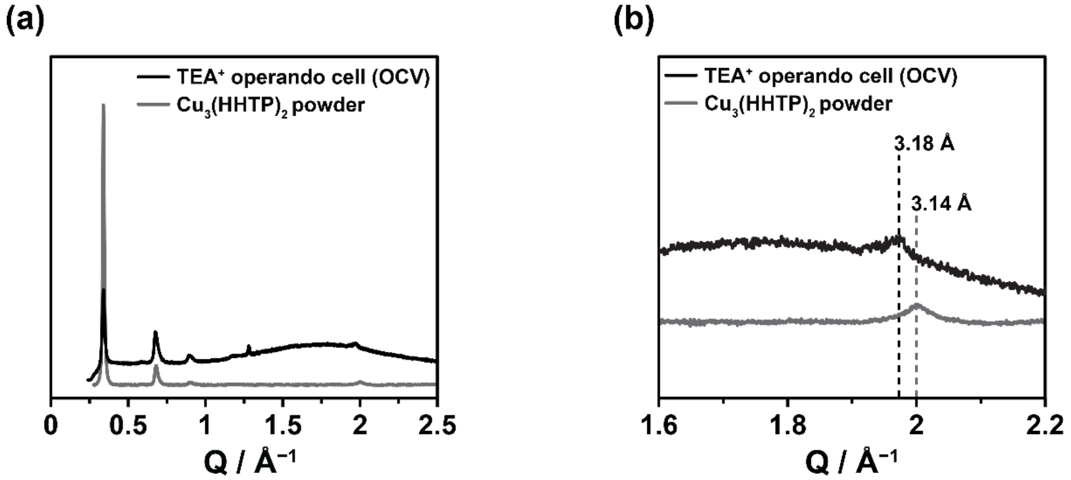

Figure S43: (a) Experimental synchrotron XRD patterns from $Cu_3(HHTP)_2$ powder (grey) and $Cu_3(HHTP)_2$ electrodes assembled in an operando symmetric two-electrode supercapacitor with 1 M TEATFSI in acetonitrile (black). (b) Zoomed-in view of the (001) reflection. The small rightward shift in the position of the (001) peak indicates an increase in the interlayer spacing of $Cu_3(HHTP)_2$ upon cell assembly with TEATFSI, consistent with wetting-induced electrode swelling.[5]

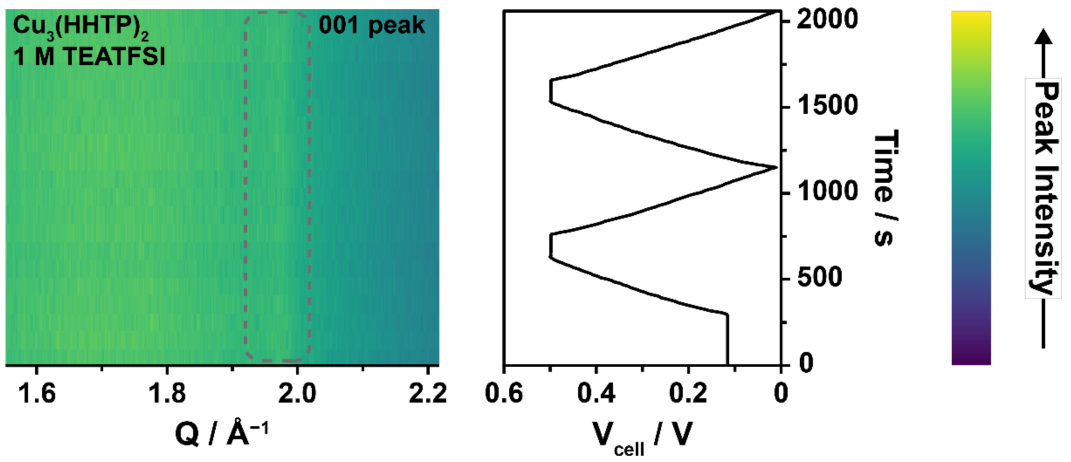

Figure S44: Operando XRD map of $Cu_3(HHTP)_2$ electrodes in a symmetric two-electrode supercapacitor assembled with 1 M TEATFSI in acetonitrile. The (001) reflection, corresponding to the interlayer spacing, is highlighted by the grey dashed box. The applied charging protocol is shown alongside and is time-aligned with the XRD data. Minimal variation in the interlayer spacing is observed during electrochemical cycling.

Additional Discussion

Paramagnetic NMR Calculations

The structure of a single $Cu_3(HHTP)_2$ layer containing a Li^+ ion with 2 or 3 acetonitrile molecules was optimised with hybrid DFT calculations to understand the coordination of alkali ions and solvent molecules with the Cu^{2+} node. The structure of the cells after optimisation are shown in SI Figure S33 below.

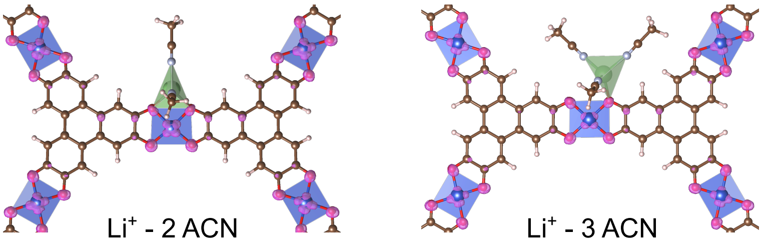

Figure S45: Hybrid DFT optimised structures of a $Cu_3(HHTP)_2$ layer containing a single Li^+ ion with 2 or 3 acetonitrile ligands. Li, Cu, C, O, H and N atoms are shown as green, blue, brown, red, white and silver, respectively. Spin density isosurface shows spin up (magenta) magnetisation density at an isosurface level of 0.02 a_0^{-3}.

For the structure containing 1 Li^+ ion and 2 acetonitrile molecules, geometry optimisation resulted in a distorted tetrahedral coordination for the Li^+ ion with one acetonitrile molecule within the $Cu_3(HHTP)_2$ plane and the other perpendicular to the plane. The Li^+ tetrahedron formed an edge sharing configuration with two oxygen ligating groups at the Cu^{2+} node, with a Cu–O–Li angle of 91.6 °. A distorted tetrahedral Li^+ configuration was also found for the structure containing 3 acetonitrile molecules, but in this case, the Li^+ ion was only connected to a single oxygen ligating group (corner sharing) at the Cu^{2+} node, with a Cu–O–Li angle of 119.1°. Two of the acetonitrile molecules were oriented within the $Cu_3(HHTP)_2$ plane, with the third molecule perpendicular to the plane.

Fermi Contact Shifts

The Fermi contact interaction on ^7Li sites from the Cu^{2+} (S = ½) node was calculated for different solvent configurations. The ^7Li Fermi contact shift results from the transfer of unpaired electron spin from the Cu^{2+} (S = ½) node, through the oxygen ligand to the Li^+ ion. The shift values for each structure are shown in SI Table S4 above.

The size and the sign of Fermi contact shifts can be rationalised based on the Goodenough Kanamori rules, as shown in previous works.[6,7] The unpaired spin on Cu^{2+} is associated with the $d_{x^2-y^2}$ antibonding orbital (e_g*) within the $Cu_3(HHTP)_2$ plane, as can be seen from the spin density isosurface in SI Figure S33. The negative shift observed for ~90 ° Cu–O–^{7}Li pathway is the result of a polarisation mechanism. For a 90 ° pathway, at the oxygen site, orthogonal O($2p_x$) and O($2p_y$) orbitals are oriented towards the Cu and Li, respectively: Cu($d_{x^2-y^2}$) – O($2p_x$) – O($2p_y$) – Li(2s). Strong overlap between the Cu($d_{x^2-y^2}$) and O($2p_x$) orbitals results in a small, positive spin density on the O($2p_x$) orbital. The positive, unpaired spin on the O($2p_x$) orbital polarises the O($2p_y$) orbital with the same positive spin, which leads to a transfer of negative spin density to the Li(2s) orbital.

A 180° Cu–O–^{7}Li within the $Cu_3(HHTP)_2$ plane would be expected to lead to a large positive shift, as positive spin density is transferred from the Cu($d_{x^2-y^2}$) via the O($2p_x$) orbital to the Li. The 119.1 ° Cu–O–^{7}Li pathway for the Li^+/3 acetonitrile system is intermediate between the negative 90 ° and positive 180 ° Cu–O–Li pathway configurations, which results in a small positive shift. Small positive shifts for Li in tetrahedral configurations are commonly seen in Li transition metal oxide cathode materials.[8]

Bibliography

(1) Xu, Z.; Mapstone, G.; Coady, Z.; Wang, M.; Spreng, T. L.; Liu, X.; Molino, D.; Forse, A. C. Enhancing Electrochemical Carbon Dioxide Capture with Supercapacitors. *Nat Commun* **2024**, *15* (1), 7851. https://doi.org/10.1038/s41467-024-52219-3.

(2) Thommes, M.; Kaneko, K.; Neimark, A. V.; Olivier, J. P.; Rodriguez-Reinoso, F.; Rouquerol, J.; Sing, K. S. W. Physisorption of Gases, with Special Reference to the Evaluation of Surface Area and Pore Size Distribution (IUPAC Technical Report). *Pure and Applied Chemistry* **2015**, *87* (9–10), 1051–1069. https://doi.org/10.1515/pac-2014-1117.

(3) *Adsorption by Powders and Porous Solids*; Elsevier, 1999. https://doi.org/10.1016/B978-0-12-598920-6.X5000-3.

(4) Shin, S.-J.; Gittins, J. W.; Golomb, M. J.; Forse, A. C.; Walsh, A. Microscopic Origin of Electrochemical Capacitance in Metal–Organic Frameworks. *J. Am. Chem. Soc.* **2023**, *145* (26), 14529–14538. https://doi.org/10.1021/jacs.3c04625.

(5) Seyffertitz, M.; Balhatchet, C. J.; Rauscher, M. V.; Stock, S.; Fritz-Popovski, G.; Leiner, T.; Holec, D.; Amenitsch, H.; Forse, A. C.; Paris, O. Selective Anion Anchoring in MOF-Based Supercapacitors Revealed with Operando Small-Angle X-Ray Scattering. *Nat Commun* **2025**, *16* (1), 8649. https://doi.org/10.1038/s41467-025-63772-w.

(6) Carlier, D.; Ménétrier, M.; Grey, C. P.; Delmas, C.; Ceder, G. Understanding the NMR Shifts in Paramagnetic Transition Metal Oxides Using Density Functional Theory Calculations. *Phys. Rev. B* **2003**, *67* (17), 174103. https://doi.org/10.1103/PhysRevB.67.174103.

(7) Genreith-Schriever, A. R.; Coates, C. S.; Märker, K.; Seymour, I. D.; Bassey, E. N.; Grey, C. P. Probing Jahn–Teller Distortions and Antisite Defects in $LiNiO_2$ with ^{7} Li NMR Spectroscopy and Density Functional Theory. *Chem. Mater.* **2024**, *36* (9), 4226–4239. https://doi.org/10.1021/acs.chemmater.3c03103.

(8) Grey, C. P.; Dupré, N. NMR Studies of Cathode Materials for Lithium-Ion Rechargeable Batteries. *Chem. Rev.* **2004**, *104* (10), 4493–4512. https://doi.org/10.1021/cr020734p.
